# Genomics‐informed delineation of conservation units in a desert amphibian

**DOI:** 10.1111/mec.16660

**Published:** 2022-08-30

**Authors:** Brenna R. Forester, Melanie Murphy, Chad Mellison, Jeffrey Petersen, David S. Pilliod, Rachel Van Horne, Jim Harvey, W. Chris Funk

**Affiliations:** ^1^ Department of Biology, Colorado State University Fort Collins CO USA; ^2^ Department of Ecosystem Science and Management, Program in Ecology University of Wyoming Laramie WY USA; ^3^ U.S. Fish and Wildlife Service Reno NV USA; ^4^ Nevada Department of Wildlife Elko NV USA; ^5^ U.S. Geological Survey, Forest and Rangeland Ecosystem Science Center Boise ID USA; ^6^ U.S. Forest Service Sparks NV USA; ^7^ Graduate Degree Program in Ecology Colorado State University Fort Collins CO USA

**Keywords:** adaptive differentiation, conservation genomics, evolutionarily significant units, genetic rescue, management units, *Rana luteiventris*

## Abstract

Delineating conservation units (CUs, e.g., evolutionarily significant units, ESUs, and management units, MUs) is critical to the recovery of declining species because CUs inform both listing status and management actions. Genomic data have strengths and limitations in informing CU delineation and related management questions in natural systems. We illustrate the value of using genomic data in combination with landscape, dispersal and occupancy data to inform CU delineation in Nevada populations of the Great Basin Distinct Population Segment of the Columbia spotted frog (*Rana luteiventris*). *R. luteiventris* occupies naturally fragmented aquatic habitats in this xeric region, but beaver removal, climate change and other factors have put many of these populations at high risk of extirpation without management intervention. We addressed three objectives: (i) assessing support for ESUs within Nevada; (ii) evaluating and revising, if warranted, the current delineation of MUs; and (iii) evaluating genetic diversity, effective population size, adaptive differentiation and functional connectivity to inform ongoing management actions. We found little support for ESUs within Nevada but did identify potential revisions to MUs based on unique landscape drivers of connectivity that distinguish these desert populations from those in the northern portion of the species range. Effective sizes were uniformly small, with low genetic diversity and weak signatures of adaptive differentiation. Our findings suggest that management actions, including translocations and genetic rescue, might be warranted. Our study illustrates how a carefully planned genetic study, designed to address priority management goals that include CU delineation, can provide multiple insights to inform conservation action.

## 1 INTRODUCTION

Appropriate identification of conservation units (CUs) is essential for effective management because CUs inform conservation listing status (e.g., under state, provincial, or federal jurisdictions) and management options available to support population recovery and persistence. Conservation unit is a general term for an array of biological and legal categories used to define subspecific population units for the protection and management of intraspecific diversity. Because inappropriate delineation of CUs can waste conservation dollars, hamper recovery efforts and result in inappropriate management actions (Weeks et al., 2016), careful evaluation of CU delineation is crucial for species of conservation concern.

One of the most commonly used CUs is the evolutionarily significant unit (ESU), which represents major components of intraspecific variability based on genetic, phenotypic, ecological, geographical and life history characteristics. There are many definitions and sets of criteria for defining ESUs (reviewed in Fraser & Bernatchez, 2001), but here we will focus on the parameters established by Waples (1991): ESUs show substantial reproductive isolation from other conspecific populations and represent an important component of the evolutionary legacy of the species. We use this definition because it best reflects the analogous policy unit under the U.S. Endangered Species Act (ESA), the “distinct population segment” (DPS). Note that DPS designation is a policy decision made by the agencies that implement the ESA, so this study will not evaluate DPSs. Management units (MUs), which are often nested within ESUs, are demographically independent, meaning that population dynamics rely on local birth and death rates rather than immigration (Palsbøll et al., 2007). MUs typically warrant separate management due to their importance for maintaining functional ecological and evolutionary processes within the larger ESU (Moritz, 1999). The conservation of multiple MUs within an ESU is considered essential for maintaining long‐term persistence, especially in species showing metapopulation dynamics (Gustafson et al., 2007; Weckworth et al., 2018).

These biologically defined CUs interface with policy definitions in diverse ways across jurisdictions. For example, under the ESA, legal protections can be extended not only to species and subspecies, but also DPSs of vertebrates. Since the ESA does not specify the basis for DPSs, the two agencies responsible for implementing the ESA have defined the term based on two main axes: “discreteness” (reproductive isolation) and “significance” (substantial contribution to evolutionary legacy and potential for persistence), similar to the Waples (1991 ESU definition above (USFWS & NMFS, 1996; Waples, 2006). Despite this clarification, CU delineation is often a complex task under the ESA, with ramifications for candidate listing determinations and extinction risk assessments (Waples & Lindley, 2018).

Genetic data can play an important role in informing the complexities of CU delineation and subsequent management and policy decision‐making. Neutral genetic markers, including mitochondrial DNA, microsatellites and single nucleotide polymorphisms (SNPs), can be used to evaluate reproductive isolation in ESUs and, to a limited extent, demographic independence in MUs (Allendorf et al., 2010; Lowe & Allendorf, 2010; Palsbøll et al., 2007). Additionally, large genome‐wide data sets have improved our ability to identify potential adaptive differentiation, informing the “evolutionary legacy” criterion in ESUs. Importantly, however, it is not always clear how to delineate CUs based solely on genetic or genomic data. For example, there is no single, simple relationship between demographic independence and genetically informed estimates of population structure (Palsbøll et al., 2007), which means that genetic data alone can often provide little information on the demographic independence criterion for MUs (Lowe & Allendorf, 2010). These limitations are important to consider in the context of management objectives for species, given the importance of CUs for listing and conservation decision‐making (Taylor & Dizon, 1999).

**FIGURE 1:**
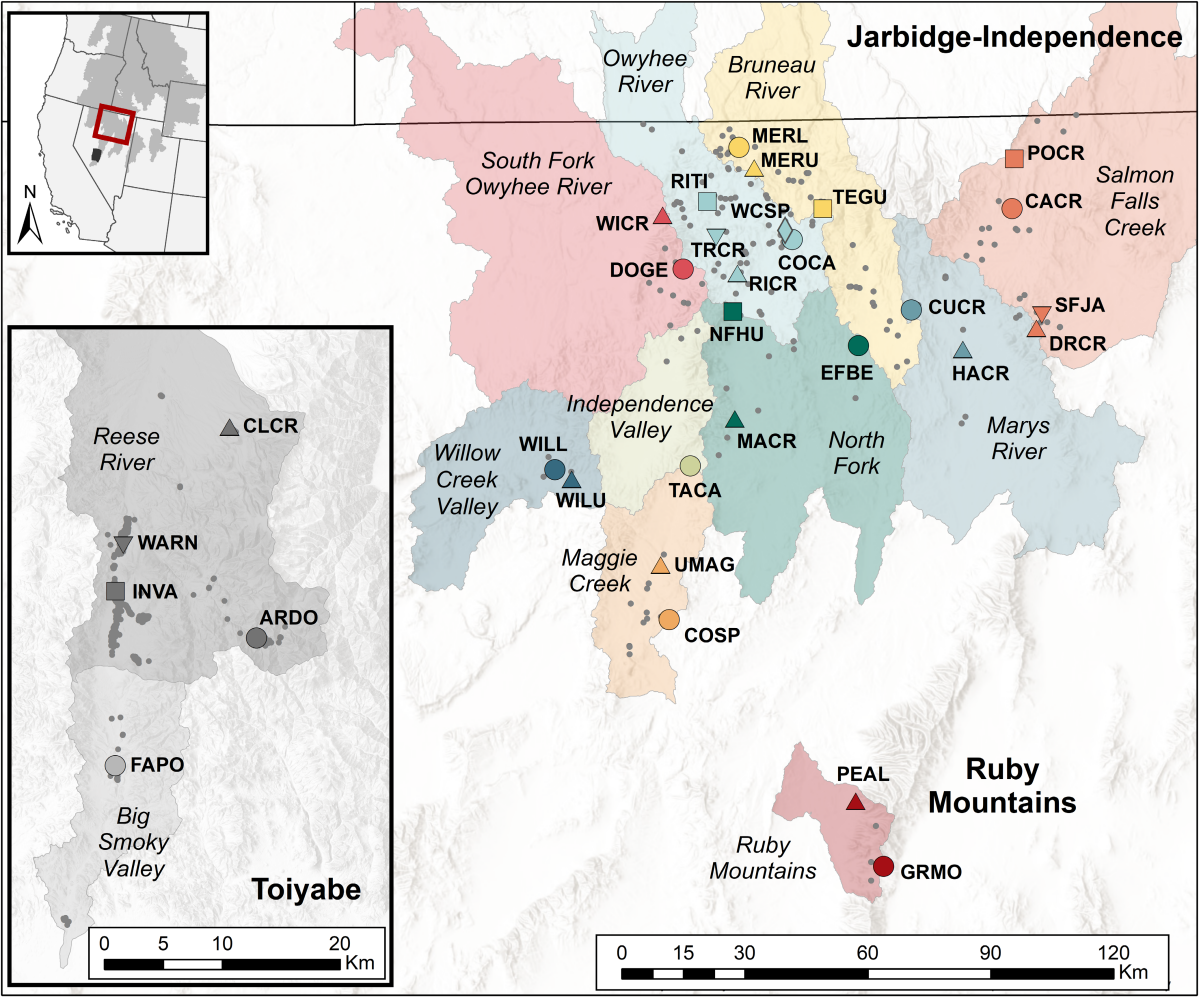
Locations of 31 *Rana luteiventris* sites sampled for genomic data in Nevada (large shapes with four‐letter bold labels). Small grey points show other known occupied sites. Sampled sites are colour coded based on watershed/current management unit (labelled in italics). Major geographical regions are labelled in bold. The Toiyabe region (inset) is disjunct from the remainder of the range and represents the southernmost locations in the species range. Extent map shows the Jarbidge‐Independence and Ruby Mountains regions in red, with the Toiyabe region in black; grey range map from the USGS Gap Analysis Project (2018).

We provide a case study in the use of genomic data to inform CU delineations in a desert amphibian. By combining multiple genomic analyses with environmental data and information on dispersal capacity and site occupancy, we evaluate evidence for ESUs and MUs in Nevada populations of the Columbia spotted frog (*Rana luteiventris*). *R. luteiventris* occupies a large range that has been divided into three major clades (Funk et al., 2008; Figure S1). We analysed genome‐wide SNP data from sites throughout Nevada, representing most of the range of the Great Basin clade of *R. luteiventris* and all three occupied regions in Nevada: the Jarbidge‐Independence (the core part of the range), the nearby but isolated Ruby Mountains and the geographically disjunct Toiyabe (Figure 1). While these populations occupy naturally fragmented aquatic habitats in this xeric region, anthropogenic activities over the past 200 years, such as beaver extirpation, stream incision, water diversions, wetland draining, agricultural irrigation and urbanization, have substantially altered the regional hydrology. These activities, along with changes in climate, have probably eliminated or altered many of the cool lentic and lotic habitats preferred by *R. luteiventris* (Arkle & Pilliod, 2015; Welch & MacMahon, 2005). Across the Great Basin, extant populations of *R. luteiventris* persist in locations where temperature and precipitation have changed the least in the last 100 years, suggesting that remaining populations may be more isolated than they were historically (Pilliod et al., 2015). Further evidence for this isolation effect was found in a survey of 220 sites across the Great Basin, which reported that watershed occupancy increased with precipitation and decreased with temperature (Smith & Goldberg, 2020). The compounding impacts of invasive species (i.e., American bullfrog [*Lithobates catesbeianus*], predatory fishes), disease, drought and climate change places many of these populations at high risk of extirpation without management intervention (Arkle & Pilliod, 2015; Mims et al., 2020; Pilliod et al., 2015, 2021).

The U.S. Fish and Wildlife Service (USFWS) first identified the Great Basin clade of *R. luteiventris* as a DPS eligible for listing under the ESA in 1997. At that time, the DPS was placed on the candidate list, meaning there was sufficient information to propose the DPS as threatened, but the proposal was precluded by higher priority listing activities. The Great Basin DPS remained on the candidate list until a “not warranted” ruling resulted in its removal in 2015 (USFWS, 1993, 2015a). Candidate listing removal was due in large part to proactive conservation actions by diverse stakeholders in the form of ongoing 10‐year Conservation Agreement and Strategy plans (Columbia Spotted Frog Technical Team, 2003a, 2003b, 2015; McAdoo & Mellison, 2017). Despite these efforts, however, many *R. luteiventris* populations in the Great Basin continue to experience declines (USFWS, 2015b). To inform potential management actions to stem declines and improve population viability, the 2015 Conservation Agreement and Strategy plan initiated a genetic study to inform the delineation of CUs and prioritize sites for ongoing conservation actions. Because Nevada populations have not been included in previous fine‐scale genetic analyses of *R. luteiventris*, current MUs are based on watershed delineations, which may not reflect actual connectivity among populations (Columbia Spotted Frog Technical Team, 2015; Figure 1). Additionally, genetic data are expected to inform other priorities, such as identifying dispersal corridors, evaluating genetic diversity within populations and adaptive differentiation among populations, and identifying suitable populations for actions such as genetic rescue (Bell et al., 2019; Frankham, 2015; Whiteley et al., 2015). These actions are especially important for the long‐term viability of patchily distributed species such as Great Basin *R. luteiventris*, which have relatively low dispersal capacity (~5–6 km observed: Funk, Greene, et al., 2005; Reaser, 1996) and small population sizes, putting them at risk of increased genetic drift, inbreeding, decreased fitness and extirpation.

To inform ongoing management of *R. luteiventris* in Nevada, we addressed three primary research questions: (i) Are there ESUs within Nevada populations of the currently defined Great Basin DPS? (ii) Do genetic data and connectivity analyses support the current delineation of MUs in Nevada? If not, how should MUs be revised to better support management goals? (iii) How should MUs, sites and movement corridors be managed and prioritized for conservation actions such as genetic rescue and reintroduction efforts?

## 2 MATERIALS AND METHODS

### 2.1 Sample collection

Toe and tail clips were collected between 2010 and 2019 from 31 sites (Figure 1). Individuals were captured at breeding ponds and released at their capture location. Sampling was conducted under authorization of USFWS (Reno, NV) and Nevada Department of Wildlife (Elko, NV).

### 2.2 Genotyping

We used Qiagen DNeasy Blood and Tissue Kits for DNA extraction, adding 4 μl of RNase after tissue digestion. We quantified DNA using Qubit dsDNA assays and verified quality in a subset of samples using agarose gel electrophoresis. We used double digest restriction‐site associated sequencing (ddRADseq) to develop genome‐wide SNPs (Peterson et al., 2012), testing five combinations of restriction enzymes to identify the combination producing fragment sizes in the target range (250–450 bp). Using *NspI* and *Eco*RI we digested ~1 μg of DNA per individual. For library production, we used a Pippin Prep size selection of 346–456 bp, KAPA HiFi Hotstart Ready Mix for polymerase chain reaction (PCR) amplification, degenerate barcodes on the P2 adapter to allow for removal of PCR duplicates (Schweyen et al., 2014), and Dynabeads cleanup to retain molecules with P1 and P2 adapters. We sequenced one library with the University of Oregon Genomics Core Facility (HiSeq‐4000, 100 bp paired‐end reads), and the remaining nine libraries with Novogene Corp. (HiSeq‐4000, 150 bp paired‐end reads). The first two libraries contained 24 and 36 individuals; all remaining libraries contained 40 individuals. Individuals from sampled locations were split across libraries to avoid confounding location and library effects.

We used fastqc v.0.11.8 (Andrews, 2019) to assess data quality. We removed PCR duplicates using *clone_filter* from stacks version 2.41 (Rochette et al., 2019). Using trim galore! version 0.6.4 (Krueger, 2019) and cutadapt version 2.5 (Martin, 2011), we removed degenerative barcodes and applied quality screening using a Phred cutoff = 20 and auto‐detect adapter screening (stringency = 6). We used stacks *process_radtags* to demultiplex, trim reads to 90 bp, remove reads with an uncalled base or low‐quality scores, and rescue barcodes and cutsites with at most one mismatch.

We used within‐ and across‐library replicates to optimize three de novo stacks parameters: minimum stack depth (‐m), distance between stacks (‐M) and distance between catalog loci (‐n). We used 19 individuals (each with a replicate sample) from 16 sites, covering the geographical range of sampling, and tested 13 combinations of parameters starting from default settings (‐m 3, ‐M 2, ‐n 1). We evaluated mean coverage, number of assembled loci, number of polymorphic loci shared across 80% of individuals, number of SNPs (Paris et al., 2017) and population/duplicate clustering using multidimensional scaling plots (Gugger et al., 2018) in plink version 1.90 (Chang et al., 2015).

We ran stacks on all unique samples (retaining the replicate with highest coverage) using the following parameters: ‐m 3, ‐M 2, ‐d, ‐‐model‐type bounded, ‐‐bound‐high 0.05, ‐n 3, ‐‐model marukilow. We applied a coarse filter using *populations*: ‐p 1, ‐R 0.3, ‐‐min_mac 2. We discarded four individuals with high missingness (>55%) before filtering with the r package *radiator* (Gosselin et al., 2020) using the following settings: retain common markers across 31 sites; retain SNPs with global minor allele count (MAC) >9; retain loci with coverage between 6 and 100, genotyped in at least 80% of individuals, and with ≤7 SNPs per locus; retain one SNP per locus, keeping the SNP with highest MAC. We removed 204 SNPs that deviated significantly (*p* < .05) from Hardy–Weinberg proportions in five or more sites (using sites with ≥10 individuals). We removed 1328 SNPs that were heterozygous in all individuals (potential paralogues) in at least one site (using sites with seven or more individuals genotyped). We removed 476 SNPs associated with a weak lane effect in the first library by identifying the contributing SNPs using redundancy analysis (RDA) from the package *vegan* version 2.5‐6 (Oksanen et al., 2019). We first imputed missing values using *snmf* in lea version 3.1.2 (Frichot & François, 2015), testing *K* = 1–15 and alpha (regularization parameter) = 10, 100 and 1000, with 25 repetitions, 200 iterations and 5% cross‐entropy withholding (final settings: *K* = 10, alpha = 10). The RDA used the imputed data set as the response and a dummy variable for library as the predictor. Using a threshold of ±4 SD from the mean loading on the constrained axis, we identified the loci contributing to the lane effect and removed them (Figure S2). We used the *whoa* package version 0.0.2.999 (Anderson, 2019) to assess heterozygote miscall rates for all sites with sample sizes ≥11, using polymorphic SNPs genotyped in at least 67%–86% of individuals (depending on sample size). We did not remove putative siblings based on recommendations from Waples and Anderson (2017). We used r versions 3.6.3 and 4.1.0 (gravity models only, R Core Team, 2020).

### 2.3 Objective 1: ESUs

We identified candidate adaptive markers using partial RDA (pRDA) at the site level (Forester et al., 2018), a genotype–environment association analysis that identifies genomic signatures of selection related to environmental variables. We removed low‐frequency SNPs (variable in <3/31 sites, retaining 38,712 SNPs) and used three uncorrelated (|*r*| < 0.6), biologically relevant environmental predictors derived from the 800‐m PRISM climate normal (1981–2010, Daly et al., 2008): average summer precipitation (June–August, when frogs are at breeding sites or moving among foraging areas), average autumn precipitation (September–November, when frogs are moving to overwintering sites) and average winter minimum temperatures (December–February, a measure of winter severity). We accounted for population structure using PC axes (Capblancq & Forester, 2021). Although the principal components analysis (PCA) screeplot (Figure S3) showed a break in variance at PC1, we retained seven PCs based on population structure results (below). We used a candidate threshold of loci loading ±3.5 SD from the mean loading of the first two constrained axes. We used a site‐based RDA of candidate markers to identify drivers of adaptive divergence and an individual‐based PCA to investigate adaptive differentiation.

We assessed reproductive isolation by evaluating population structure using neutral markers (genetic data with candidate adaptive SNPs removed). We calculated pairwise *F*_ST_ (Weir & Cockerham, 1984) using stampp version 1.6.3 (Pembleton et al., 2013) and visualized population structure of the individual‐based data using a model‐free approach, PCA, and an ancestry estimation method, admixture version 1.3 (Alexander et al., 2009). For PCA, we used the *snmf*‐imputed data. For admixture, we tested *K* = 2–20 and used a cross‐validation plot to determine the best *K*(s).

### 2.4 Objective 2: MUs

We used multiple analyses to evaluate MUs including population structure analyses as above. We evaluated functional connectivity in the Jarbidge‐Independence among genetic sites (*n* = 24) and unsampled but occupied sites (*n* = 170; Naujokaitis‐Lewis et al., 2013) using predictions from gravity models set to a 15‐km maximum distance (full methods below). We selected 15 km based on the longest multiyear dispersal event from an extensive capture–recapture data set collected in the Toiyabe Mountains in Nye County, Nevada, from 1996 to 2021 (D. S. Pilliod and others, unpublished data). This study was conducted in the upper Reese River and all upper tributaries, as well as Cloverdale Creek in the Big Smoky Valley, an area spanning about 500 km^2^ (~34 km north–south and 16 km east–west). A total of 15,990 adults (>45‐mm snout–vent length) were marked using passive integrated transponder (PIT) tags injected into the dorsal subcutaneous tissue during annual, multipass surveys of all wetlands over a 26‐year period (Pilliod et al., 2021). Movements were recorded as Euclidean distance of individually marked frogs and could span several years. Frogs in the study area live up to 14 years, but the majority live about 7 years on average (Cayuela et al., 2021). Occupancy data across 1355 sites in the Jarbidge‐Independence are derived from surveys conducted between 2001 and 2020 (Nevada Department of Wildlife, unpublished data). Finally, we evaluated hierarchical structure in the neutral data (pruned to markers with <5% missingness) using AMOVA in *poppr* (Kamvar et al., 2015) across current MUs, watershed units, admixture assignments and assignments based on combined results (e.g., admixture, PCA and functional connectivity).

### 2.5 Objective 3: Site evaluations and functional connectivity:

We calculated *H*_O_, *H*_E_ and nucleotide diversity from neutral markers using stacks. We estimated effective population size (*N*_e_) for sites with sample sizes ≥11 using the LD method of neestimator version 2.01 (Do et al., 2014). For *N*_e_, we pruned site‐level data to include neutral, polymorphic SNPs genotyped in at least 80% of individuals, excluding alleles with frequencies less than two critical values, 0.05 and 0.10. We corrected *N*_*e*_ and jackknife confidence intervals based on 13 chromosome pairs in *R. pretiosa* (Haertel et al., 1974) to reduce bias in *N*_e_ estimates due to retaining linked loci, which can have a larger effect on *N*_e_ estimates than either population size or sample size (Waples et al., 2016). We evaluated adaptive differentiation as in Objective 1.

We investigated landscape factors influencing functional connectivity in the Jarbidge‐Independence using gravity models (Fotheringham & O'Kelly, 1989; Murphy et al., 2010) implemented in *genetit* version 0.1‐3 (Evans & Murphy, 2020). Gravity models have three components: a measure of spatial proximity (i.e., distance, *w*), characteristics of the sampled sites that could produce migrants contributing to gene flow (at‐site characteristics, *v*) and characteristics of the landscape matrix restricting or facilitating gene flow between sites (between‐site characteristics, *c*; Table 1). We used (1 – Nei's *D*) as our measure of gene flow, calculated from the neutral SNPs using *adegenet* version 2.1.3 (Jombart & Ahmed, 2011). To estimate a singly constrained gravity model accounting for nonindependence of the pairwise genetic matrix, we took the natural log of all dependent and independent variables to linearize the equation and applied a mixed effects model (Murphy et al., 2010). We treated sites as random effects (or grouping factors), accounting for nonindependence in the matrix (Robertson et al., 2018). We sampled the landscape matrix between sites by a straight edge between locations; these edges act as a sample of the landscape and do not represent movement paths. To determine if these edges sufficiently captured environmental variation, we tested multiple edge widths (30 m native cell size, 90, 150, 270 and 510 m) and compared pairwise correlations in independent parameter values between edge widths (Watts et al., 2015). Independent parameter values were highly correlated across edge widths (*r* > 0.85); therefore, we used the native resolution value (30 m) in all subsequent analyses.

**TABLE 1:** Description of parameters used in gravity models to explain functional connectivity in *Rana luteiventris*

Parameter	Process(es)	Variable	Abbreviation	Description	Justification
Distance	Isolation by distance	Geographical distance	dist	Straight‐line geographical distance between sites (m)	Terrestrial stage frogs move overland and are dispersal‐limited (Funk, Greene, et al., 2005; Murphy et al., 2010; Pilliod et al., 2015)
At‐site	Topography, temperature	Heat load index	hli	A measure of incident radiation	A proxy for topographic exposure; influences water temperature and primary productivity; high levels reduce productivity in desert regions (Pilliod et al., 2015)
	Topography	Relative slope position	rsp	Position of a point relative to the ridge and valley, based on its elevation	Site productivity is higher closer to ridges in desert regions (Pilliod et al., 2015) and high areas may be inaccessible (Murphy et al., 2010)
	Moisture	Percent wetland	p_wetland	Percent wetland in a 165‐m radius around each site	More wetland habitat around a site may increase productivity and facilitate movement (Munger et al., 1998; Pilliod et al., 2015)
	Moisture; spatial proximity	Wetland betweeness	wetland_ betweenness	Wetlands within a network facilitate stepping stone connectivity	The spatial arrangement of wetlands influences overall site productivity (Fortuna et al., 2006; Pilliod et al., 2015)
	Moisture; spatial proximity	Wetland centrality	wetland_ centrality	Connectedness within a larger network may facilitate long‐term stability of a wetland	The spatial arrangement of wetlands influences overall site productivity (Fortuna et al., 2006; Pilliod et al., 2015)
	Source populations; spatial proximity	Betweeness, centrality and degree for occupied sites	ralu_ betweenness, ralu_centrality, ralu_degree	Connectedness to occupied sites may increase the number of potential migrants	The spatial arrangement of wetlands influences overall site productivity (Fortuna et al., 2006; Pilliod et al., 2015)
Both at‐ and between‐site	Moisture	Compound topographic index	cti	Measure of wetness based on topography (i.e., upslope moisture contribution and ability to hold moisture)	Consistently wet at‐site conditions have greater productivity; wetter intervening habitat should facilitate connectivity (Munger et al., 1998; Pilliod et al., 2015)
Between‐site	Physiologically relevant distance	Stream distance	d_stream	Distance between sites following stream corridors (m)	Frogs may be more likely to travel along riparian corridors in desert landscapes (Pilliod et al., 2015)
	Source populations; spatial proximity	Distance to occupied site	d_occupied	Straight‐line geographical distance to closest occupied site (m)	Some sites are more productive because they are closer to other productive sites
	Physiologically relevant distance	Wetland network	wetnet	Distance to closest wetland (m)	Nearby wetlands may provide stop‐over points during migration or opportunities for stepping‐stone migration (Munger et al., 1998; Pilliod et al., 2015)
	Topography; microclimate	Surface relief ratio	srr3, srr27	Surface relief ratio at 3‐m and 27‐m window sizes derived from a 30‐m digital elevation model	Fine‐ and coarse‐scale topographic complexity may facilitate/impede connectivity due to microclimates/barrier effects
	Temperature	Mean temperature of the warmest month	stemp	Average temperature of the warmest month (1961–1990)	In desert systems, high summer temperatures may limit movement due to desiccation risk and physiological costs (Pilliod et al., 2015)
	Moisture	Autumn (fall) precipitation	fprecip	Mean annual precipitation (mm) for September and October	Higher autumn precipitation may facilitate movement to overwintering sites (Pilliod et al., 2015)
	Anthropogenic effects	Percent impervious surfaces	p_impervious	Median impervious surface (roads, rocks, impervious gravel) between sites (%)	Impervious surfaces and other anthropogenic development will impede connectivity

We tested 11 hypotheses of functional connectivity (Table 2). We calculated compound topographic index (Moore et al., 1993), heat load index (McCune & Keon, 2002), relative slope position (De Reu et al., 2013) and surface relief ratio (Pike & Wilson, 1971; data from SRTM digital elevation model, USGS) using *spatialeco* version 1.3‐2 (Evans, 2021), and percentage wetland using *landscapemetrics* version 1.5.3 (Hesselbarth et al., 2019). Temperature and precipitation metrics were calculated from Rehfeldt (2006) and impervious surfaces were calculated from Dewitz (2020). To assess the spatial context of sampled sites within a larger landscape containing additional wetlands, we used Delauney triangulation (*deldir* version 0.2‐10; Turner, 2021) to create two networks: (i) all wetlands within the study area (*n* = 1682; Saito et al., 2020; Wetland Map of Nevada version 1.0), and (ii) all wetlands occupied by *R. luteiventris* between 2001 and 2020 (*n* = 194).

**TABLE 2:** Eleven hypotheses of *Rana luteiventris* functional connectivity in the Jarbidge‐Independence region and their support based on the Akaike Information Criterion corrected for small sample sizes (AICc), Bayesian Information Criterion (BIC), log likelihood and delta AICc (ΔAICc). Results for the global model are shown in italics because the model did not converge. The top model is shown in bold

Hypothesis	At‐site parameter(s)	Between‐site parameter(s)^a^	Number of parameters	AICc	BIC	Log likelihood	ΔAICc
Global^b^	All uncorrelated at‐site	All uncorrelated between‐site	14	*−261.59*	*−203.29*	*147.80*	*−1.37*
**Temperature + Moisture**	**cti, hli**	**fprecip, cti, stemp**	**6**	*−* **260.22**	*−* **229.35**	**139.11**	**0.00**
Temperature	hli	stemp	3	*−*253.21	*−*232.63	132.60	7.01
Moisture	cti	fprecip, cti	4	*−*251.19	*−*227.18	132.59	9.03
Topography	rsp	srr3, srr27	4	*−*222.59	*−*198.58	118.29	37.63
Water connectivity	wetland_betweenness, wetland_centrality	d_stream	4	*−*222.15	*−*201.57	117.07	38.07
Productivity	hli, p_wetland	—	3	*−*220.88	*−*200.30	116.44	39.34
Null (distance only)	—	—	1	*−*220.04	*−*206.32	114.02	40.18
Wetland	p_wetland, cti	—	3	*−*218.42	*−*197.84	115.21	41.80
Source populations	ralu_betweenness, ralu_centrality, ralu_degree	d_occupied	5	*−*215.65	*−*191.65	114.83	44.57
Anthropogenic	—	p_impervious	2	*−*215.39	*−*195.39	112.29	44.83

^a^ All models include distance (dist).

^b^ Did not converge.

To measure graph‐based connectivity (*igraph version* 1.2.6; Csardi & Nepusz, 2006) of wetlands and occupied sites, we calculated degree (Diestel, 2005), betweenness centrality (Brandes, 2001; Freeman, 1978) and alpha centrality (Bonacich & Lloyd, 2001) for each node for each network. For all continuous variables, we calculated the median value along the edge for each of the landscape variables (e.g., Watts et al., 2015). We tested for correlated variables using a threshold of |*r*| < 0.70.

Previous work has shown that *R. luteiventris* exhibits strong isolation‐by‐distance (IBD; Funk, Blouin, et al., 2005; Murphy et al., 2010; Robertson et al., 2018) making geographical distance the most appropriate null model (Table 2). In addition, spatial autocorrelation is expected in dispersal‐limited species such as *R. luteiventris* and is a condition of the gravity model; therefore, IBD (“distance”) is included in all hypotheses. We tested pruned networks at 50 km (the minimum distance to connect the graph across all sites), 100 km maximum distance and saturated (all edges) between observations. Because model results were similar, we present 50‐km results as they are more biologically relevant. We used the corrected Akaike information criterion (AICc) for comparison of models fit using maximum likelihood (Akaike, 1973; Burnham & Anderson, 2002). After identifying top model(s), we used restricted maximum likelihood for parameter estimates; if the confidence interval excluded zero, that parameter was considered an important driver of the model (Zuur et al., 2009).

To assess functional connectivity including occupied but unsampled sites (*n* = 170), we predicted the final model (*gravity.predict*, population level) to all wetlands with at least one documented observation between 2001 and 2020 (*n* = 194). To collect covariate data included in the final model, we used the methods above. We then predicted flow at the population level to a 15‐km maximum distance graph and a saturated (all edges) graph. We back‐transformed the natural log flow predictions using the Naihua correction (Duan, 1983).

## 3 RESULTS

### 3.1 Genotyping

We sequenced 361 individuals from 31 sites (Figure 1; Table S1). PCR duplicate rates averaged 43% (median = 41%, range = 37%–61%). After filtering, the final data set included 39,914 SNPs genotyped in 357 individuals with overall missingness of 7.6% and average depth of coverage of 29.2× (median = 30.22×, range = 7.0–47.8×). All sites had 12 individuals, except EFBE (*n* = 14), WILU and UMAG (*n* = 11), POCR (*n* = 8), RITI (*n* = 7) and SFJA (*n* = 6). The correlation between *H*_O_ and missingness was low (*r* = 0.087). Heterozygote miscall rates were low (mean = 0.010, median = 0.001, range = 0.00004–0.048, Table S2).

### 3.2 Objective 1: ESUs

**FIGURE 2:**
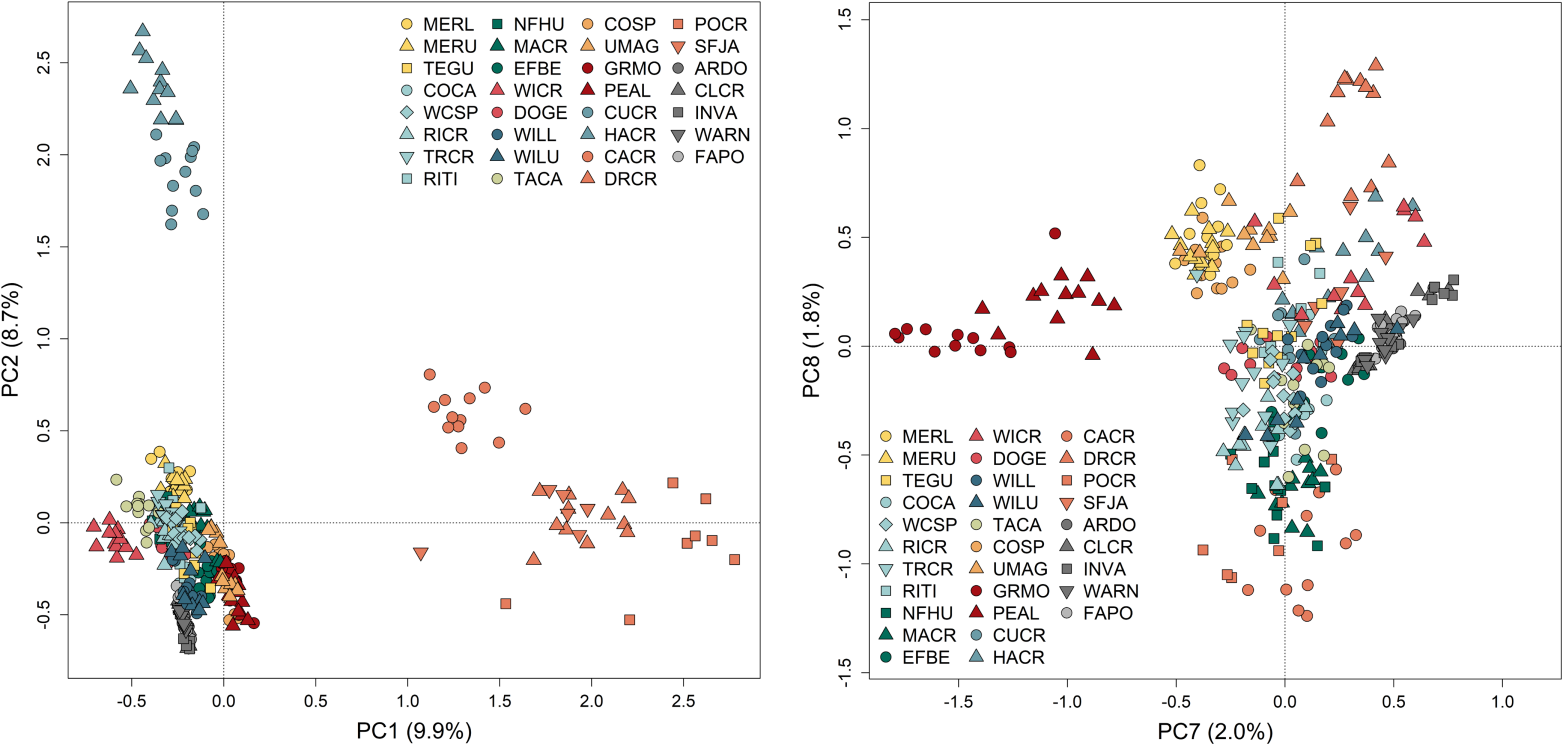
Individual‐based PCA of 689 candidate adaptive markers identified by partial redundancy analysis (colours match watersheds in Figure 1). PC1 and PC2 (left) show adaptive divergence in the Salmon Falls sites and Marys River sites, respectively. A signature of adaptive divergence in the Ruby Mountains is not identified until PC7 (right); there is no clear signature from Toiyabe sites in the top 10 PC axes.

We identified 689 candidate adaptive markers on the first two pRDA axes. Most detections were related to precipitation (240 and 222 candidates most strongly correlated with summer and autumn precipitation, respectively), with 227 candidates most strongly correlated with winter minimum temperature. There were signatures of adaptive divergence across Nevada, with Salmon Falls sites showing differentiation related to high summer precipitation, and Marys River sites related to low winter temperatures. (Figure 2; Figure S4). WICR in the South Fork Owyhee showed a weaker adaptive signature related to high autumn precipitation and high winter minimum temperatures (Figure S4). PCA of the 689 candidate adaptive markers did not reveal adaptive divergence associated with the Ruby Mountains until PC7, explaining 2.0% of the genetic variance (Figure 2), and there was no signature of adaptive divergence at the Toiyabe sites in the top 10 axes.

**FIGURE 3:**
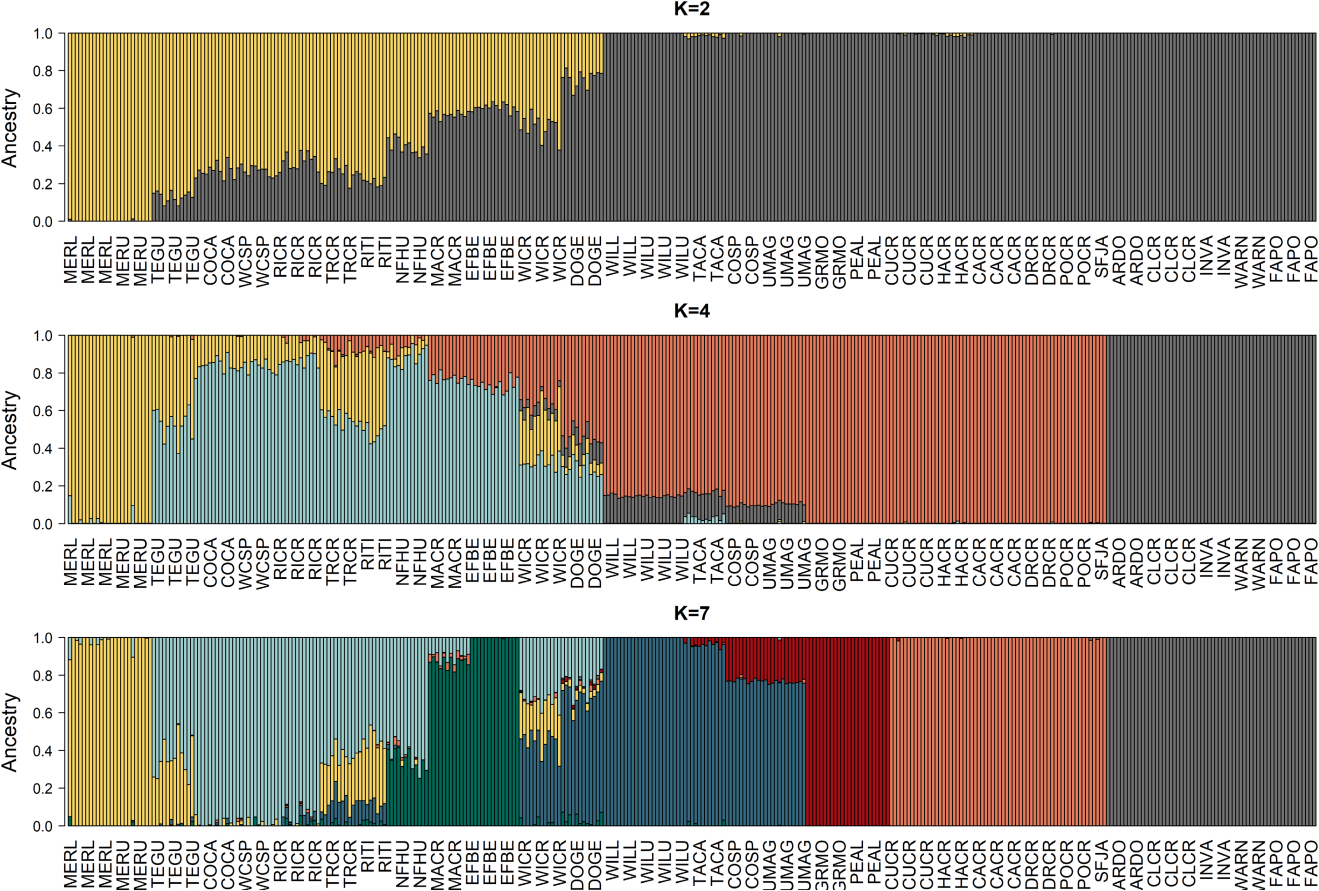
Admixture assignment plots using 39,225 neutral SNPs in 357 individuals distributed across 31 sampling sites (colours match primary watersheds in Figure 1). *K* = 2 assignments identify IBD signatures in the Jarbidge‐Independence subpopulations. The Toiyabe subpopulation is first identified at *K* = 4, while the Ruby Mountains subpopulation is first identified at *K* = 7.

**FIGURE 4:**
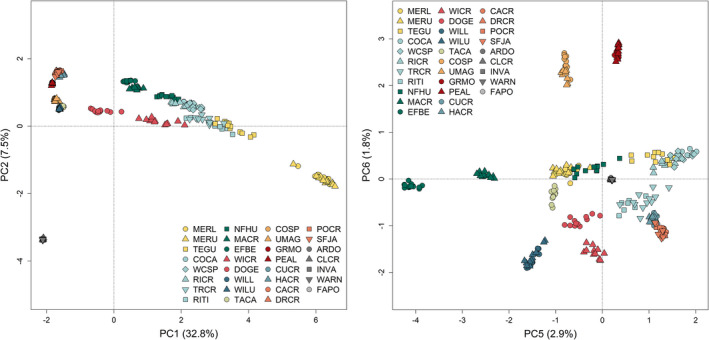
PCA of 39,225 neutral SNPs in 357 individuals distributed across 31 sampling sites (colours match primary watersheds in Figure 1). PC1 primarily identifies the IBD signatures in the core part of the Jarbidge‐Independence subpopulation, while PC2 distinguishes the geographically disjunct Toiyabe subpopulation. The Ruby Mountains subpopulation is not clearly distinguished until PC6, along with the Maggie Creek sites.

Reproductive isolation was supported in the geographically disjunct Ruby and Toiyabe regions based on neutral pairwise *F*_ST_ values, which was very high between the Ruby Mountains and all other sites (mean pairwise *F*_ST_ = 0.66) and the Toiyabe and all other sites (mean pairwise *F*_ST_ = 0.75, full matrix: Table S3). Population structure analyses identified these regions as distinct, although they were not the primary drivers of genetic divergence among Nevada sites; that is, they did not drive differentiation at the lowest values of *K* in admixture (Figure 3) or the primary PC axis in PCA (Figure 4).

### 3.3 Objective 2: MUs

**FIGURE 5:**
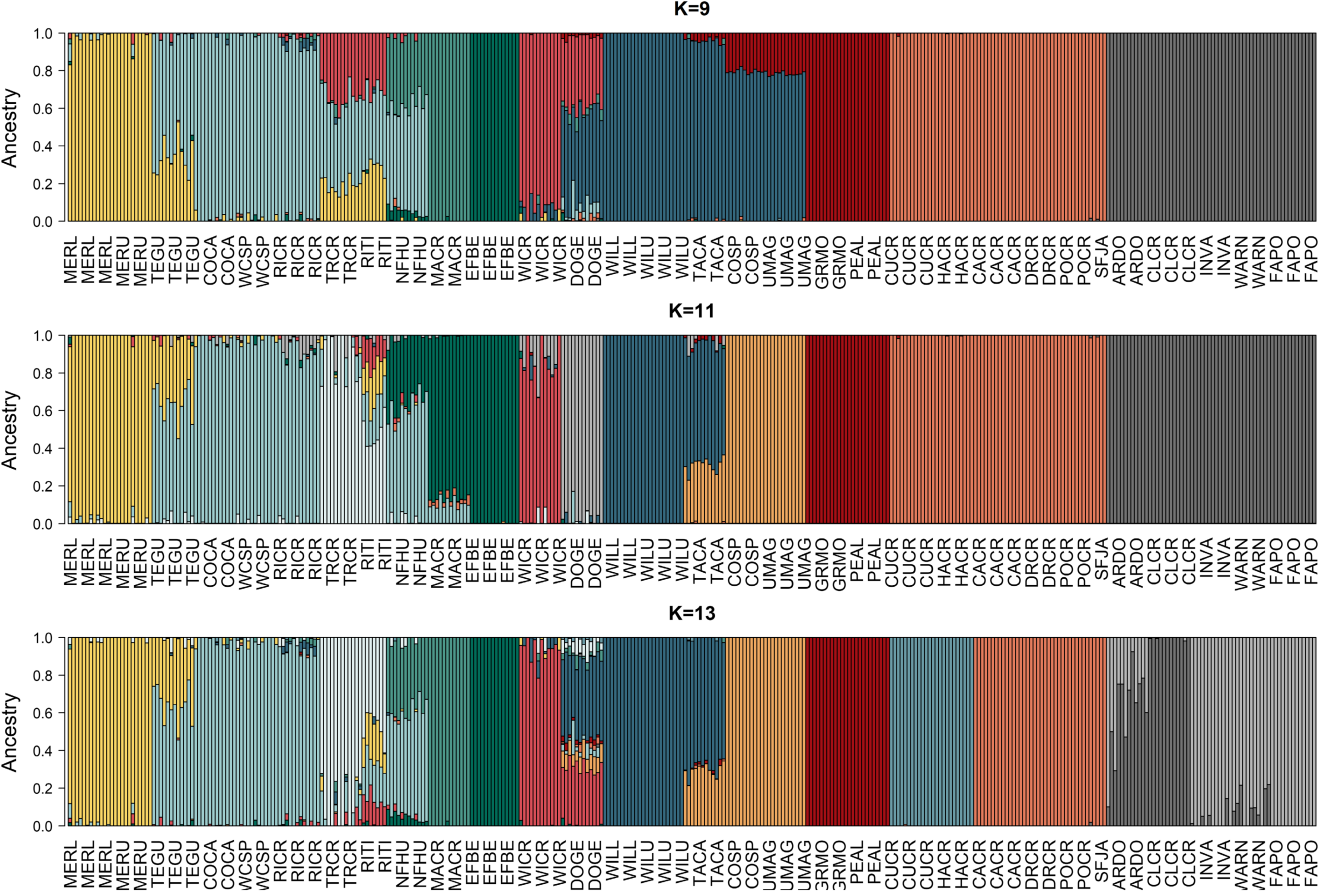
admixture assignment plots using 39,225 neutral SNPs in 357 individuals distributed across 31 sampling sites. Colours match primary watersheds in Figure 1, except for light green in *K* = 9 and 13, white in *K* = 11 and 13, and light grey in *K* = 11, which indicate genetic differentiation not related to watershed delineation.

The “best” values of *K* for admixture and the PCA screeplot were not definitive in the neutral data set (Figure S3 and S5), due in large part to IBD in the Jarbidge‐Independence region (Figures 3 and 4). admixture assignments for *K* = 9, 11 and 13 illustrate the complex relationships among watersheds (Figure 5; Figure S6, results for *K* = 8–13 in Figures S7 and S8). Consistencies across *K* values include: clear delineation of Toiyabe and Ruby regions from other areas; clear delineation of MERL and MERU in the Bruneau, with TEGU showing admixture with Owyhee sites; clear admixture of NFHU with Owyhee sites; and consistent delineation of Willow Creek and EFBE (North Fork) sites. Differences across values of *K* probably relate to differing levels of past (or possibly contemporary) gene flow. These include assignment of the Owyhee sites TRCR and RITI as either admixed or distinguished as separate groups, splitting of North Fork sites into different groups, identification of DOGE as a separate group, and the separation of Maggie and Marys sites from larger watershed groupings. Variation in assignments among Toiyabe sites are highly variable (Figures S7 and S8) and do not appear to be ecologically meaningful. Although pairwise *F*_ST_ values are high (Table S3), small effective sizes (below) mean that divergence due to drift will require a proportionally larger number of migrants to counteract, in comparison with populations with larger effective sizes (Mills & Allendorf, 1996).

**FIGURE 6:**
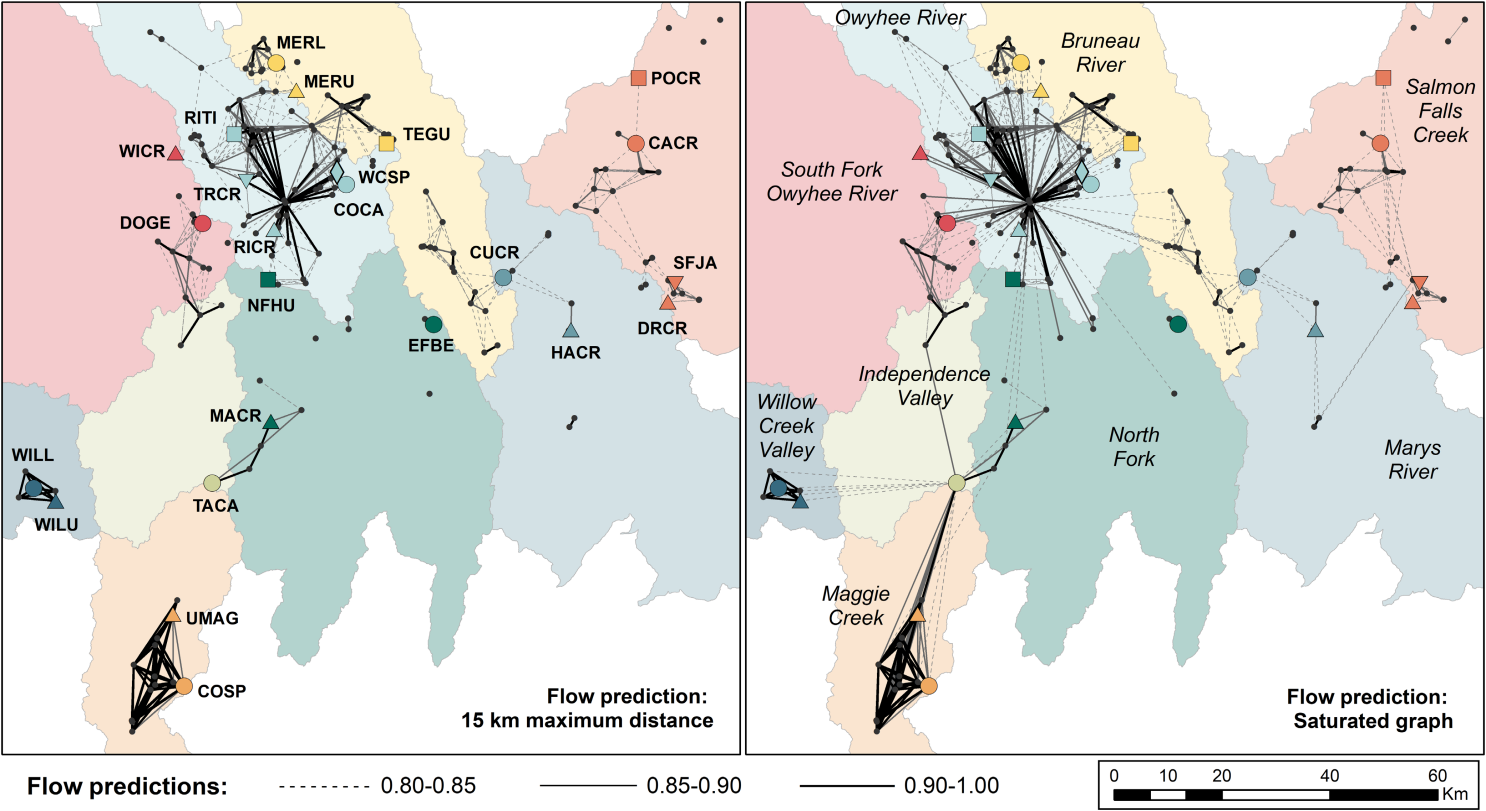
Predicted flow across sampled (coloured shapes) and unsampled but occupied (black points) sites in the Jarbidge‐Independence subpopulation. Edges shown in both panels are limited to those with predicted flow ≥0.85 (where flow = 1 − predicted Nei's *D*). The left panel shows flow between sites that are ≤15 km apart (based on maximum estimated single generation dispersal capacity); the right panel shows the saturated graph (flow between all sites). Genetic sampling locations are labelled on the left and current management units are labelled on the right.

The best‐supported model of functional connectivity was the temperature–moisture model (Table 2, additional details below, Objective 3). To better understand how sampled and unsampled sites influenced functional connectivity in the Jarbidge‐Independence, we used this top model to predict flow (defined as 1 − predicted Nei's *D*) across all occupied sites (*n* = 194) using both a 15‐km maximum distance and a saturated graph (all edges included). In both cases, we visualized flow (Figure 6) using a cutoff of 0.85 (1 – Nei's *D*, equivalent to a predicted Nei's *D* = 0.15) based on the flow prediction (0.852) for the sites with the lowest pairwise *F*_ST_ in the Jarbidge‐Independence region (WCSP–COCA, pairwise *F*_ST_ = 0.051). Flow predictions indicate that functional connectivity is limited across most of the Jarbidge‐Independence, especially peripheral areas. For example, the Salmon Falls, Willow and Maggie Creek sites show no (15 km) to limited (saturated) flow outside their watersheds at the above cutoffs, though admixture results indicate signatures of past connectivity with neighbouring sites. By contrast, flow predictions in the core part of the region show functional connectivity across watershed boundaries, supporting inference from admixture and PCA plots. In particular, TEGU (Bruneau) and NFHU (North Fork) show signatures of gene flow with Owyhee sites.

**FIGURE 7:**
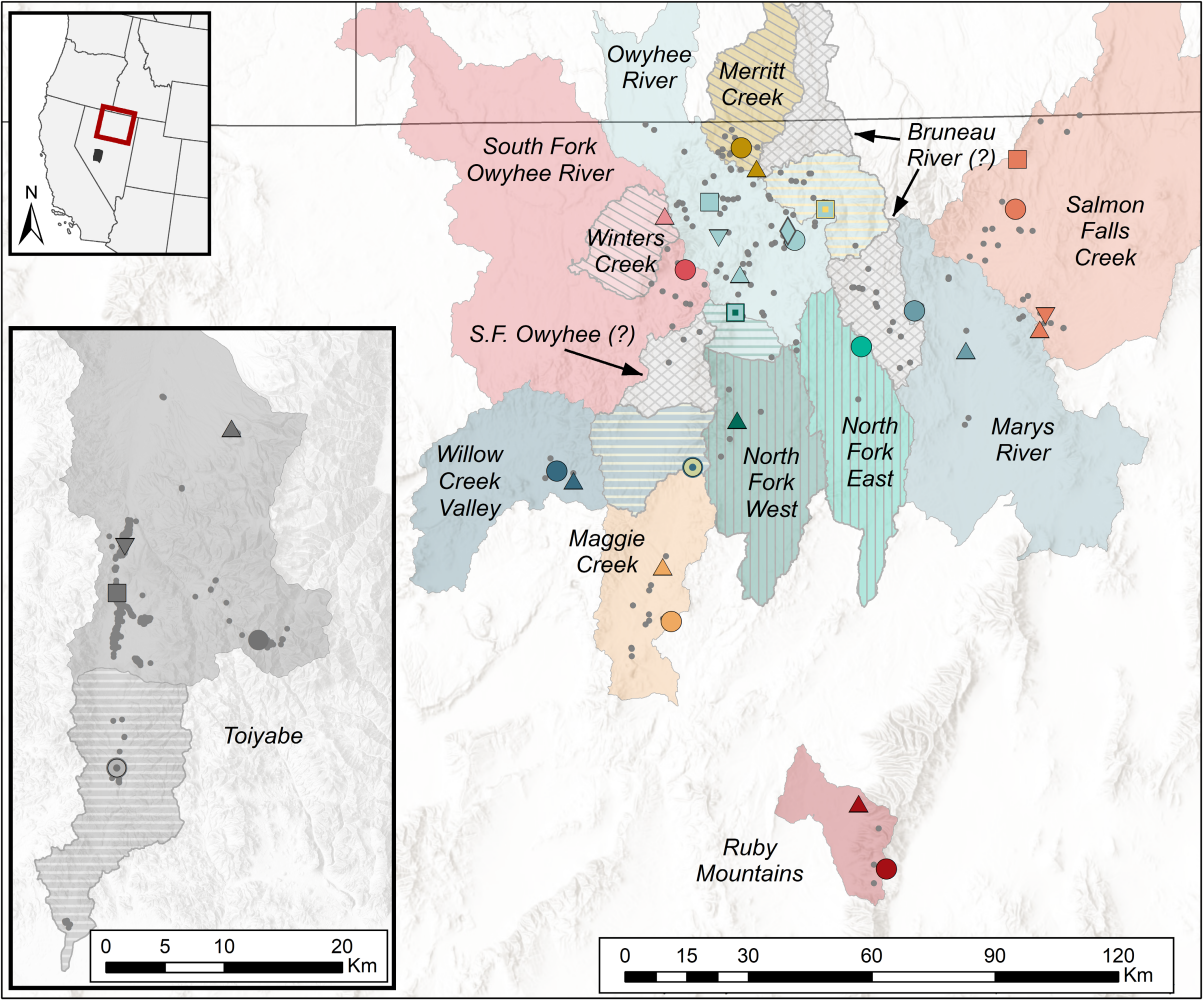
Possible reassignment of management units for *Rana luteiventris* populations in Nevada. Suggested MUs are labelled in italics. New MUs are shown with diagonal grey lines within their original watershed‐based MUs (Merritt Creek and Winters Creek); areas merged into existing MUs are shown with horizontal lines representing their new and old MU delineation; old MU delineations that have been split are shown with vertical grey lines; unassigned units are shown in grey with diamond hatching (e.g., Bruneau River). Genomic site colours/shapes follow Figure 1; dual‐colour squares and circles represent genomic sites that were reassigned to a new/different MU.

The genetic site groupings with strongest AMOVA support were delineation using HUC10 watersheds, followed by two assignments based on integration of all results (i.e., admixture, PCA, flow predictions, Table 3; Table S4). These assignments improved among‐group variance explained by ~5% over the current watershed‐based MUs. Although HUC10 delineation explained the most among‐group variance, this assignment produced 19 groups for 31 sites, creating many singleton sites (Figure S9). By comparison, assignment into 12 groups based on combined results (Figure 7; Figure S10) explained only 0.1% less among‐group variance. The three combined assignments that had 11 groups (Table 3; Table S4) used the 12‐group combined assignment (Figure 7) with the following changes: combining Salmon and Marys watersheds (“11 MUs–Salmon+Marys”), no new Winters Creek MU (“11 MUs–SF Owyhee”) and no split of the North Fork MU (“11 MUs–North Fork”).

**TABLE 3:** Management unit delineations with strongest AMOVA support.

Groups	No. of groups	Variance components	% of variation
HUC10	19	Among groups	65.8
		Among sites	6.9
		Within sites	27.3
Combined assignment, 12 MUs	12	Among groups	65.7
		Among sites	8.0
		Within sites	26.3
Combined assignment, 11 MUs – Salmon + Marys	11	Among groups	65.6
		Among sites	8.4
		Within sites	26.0
Admixture *K* = 11	11	Among groups	65.1
		Among sites	8.7
		Within sites	26.2
Combined assignment, 11 MUs – SF Owyhee	11	Among groups	65.0
		Among sites	8.8
		Within sites	26.3
Admixture K = 13	13	Among groups	64.7
		Among sites	8.6
		Within sites	26.7
Combined assignment, 11 MUs – North Fork	11	Among groups	64.7
		Among sites	9.1
		Within sites	26.3
Current (watershed‐based) MUs	12	Among groups	61.0
		Among sites	12.2
		Within sites	26.8

### 3.4 Objective 3: Site evaluations and functional connectivity

Effective population sizes were uniformly small, with an average point estimate of 30.2 (median 22.8, range 3.0–84.7, Table 4; Table S5). *N*_e_ varied across sites within MUs; for example, only two of the four Reese MU sites (in the Toiyabe) were among the smallest estimates. Neutral diversity varied widely (Table 5), with the highest levels in the core Jarbidge‐Independence, and lower diversity in the periphery. The Ruby Mountains and Toiyabe had the lowest genetic diversity, with Ruby having a large number of private alleles, indicating little gene flow between sites. There were some signatures of adaptive divergence, reported above (Objective 1).

**TABLE 4:** Effective population size (*N*_e_) estimates using a maximum allele frequency of 0.05 (i.e., Pcrit = .05). *N*_e_ and jackknife confidence intervals (CIs) are corrected for the number of chromosome pairs in *Rana pretiosa*. The “inf” estimate indicates that the confidence interval includes infinity.

Site	Management unit	Number of loci	Number of individuals	Corrected *N*_e_	Lower CI	Upper CI
MERL	Bruneau	10,000	12	22.7	9.2	inf
MERU		10,000	12	25.0	15.1	55.7
TEGU		10,000	12	42.4	17.2	inf
COCA	Owyhee	10,000	12	28.8	9.4	inf
RICR		10,000	12	55.2	27.4	547.3
TRCR		10,000	12	26.1	10.3	inf
WCSP		10,000	12	66.7	34.7	406.6
DOGE	South Fork Owyhee	10,000	12	84.7	32.5	inf
WICR		10,000	12	20.6	7.5	inf
CACR	Salmon Falls	4860	12	8.2	2.6	43.8
DRCR		2718	12	17.2	8.5	61.5
CUCR	Marys	6229	12	14.9	4.9	inf
HACR		5848	12	36.5	20.8	106.6
EFBE	North Fork	10,000	14	15.3	9.2	30.8
MACR		10,000	12	13.1	4.3	150.7
NFHU		10,000	12	22.9	8.0	inf
TACA	Independence	9122	12	83.2	38.2	inf
COSP	Maggie	3257	12	11.5	4.6	46.0
UMAG		4487	11	58.9	19.6	inf
WILL	Willow	4495	12	9.0	3.3	23.4
WILU		4550	11	32.9	15.7	309.1
GRMO	Ruby	3190	12	17.1	6.6	270.1
PEAL		1996	12	14.4	5.3	173.9
ARDO	Reese	771	12	49.0	25.1	304.3
CLCR		1057	12	10.0	5.7	19.6
INVA		814	12	12.7	6.0	39.4
WARN		675	12	44.7	16.4	inf
FAPO	Big Smoky	698	12	3.0	2.1	11.6

**TABLE 5:** Genetic diversity metrics for sampled sites.

Site	Management unit	Private alleles	Observed heterozygosity	Expected heterozygosity	Nucleotide diversity
MERL	Bruneau	1	0.219	0.216	0.0013
MERU		3	0.216	0.200	0.0012
TEGU		1	0.267	0.271	0.0016
COCA	Owyhee	0	0.244	0.241	0.0014
RICR		0	0.243	0.240	0.0014
RITI		0	0.302	0.284	0.0017
TRCR		2	0.300	0.282	0.0016
WCSP		0	0.244	0.237	0.0014
DOGE	South Fork Owyhee	7	0.191	0.189	0.0011
WICR		4	0.283	0.265	0.0015
CACR	Salmon Falls	10	0.045	0.043	0.0003
DRCR		4	0.030	0.027	0.0002
POCR		0	0.040	0.036	0.0002
SFJA		0	0.037	0.033	0.0002
CUCR	Marys	13	0.050	0.049	0.0003
HACR		11	0.060	0.056	0.0003
EFBE	North Fork	34	0.145	0.135	0.0008
MACR		8	0.196	0.181	0.0010
NFHU		1	0.248	0.245	0.0014
TACA	Independence	13	0.086	0.083	0.0005
COSP	Maggie	31	0.048	0.044	0.0003
UMAG		21	0.054	0.053	0.0003
WILL	Willow	3	0.064	0.059	0.0003
WILU		1	0.065	0.062	0.0004
GRMO	Ruby	56	0.028	0.027	0.0002
PEAL		51	0.021	0.021	0.0001
ARDO	Reese	0	0.006	0.005	0.00003
CLCR		3	0.006	0.006	0.00003
INVA		0	0.006	0.005	0.00003
WARN		0	0.005	0.004	0.00003
FAPO	Big Smoky	0	0.005	0.005	0.00003

The temperature–moisture connectivity model included the following parameters with 95% confidence intervals that did not overlap zero (Table 6): distance, autumn precipitation, mean temperature of the warmest month, compound topographic index (cti) at and between sites, and heat load index. Geographical distance between sites was negatively correlated with gene flow, supporting IBD. Both increased cti and autumn precipitation between sites contributed positively to gene flow, whereas higher mean temperatures of the warmest month decreased gene flow. A high heat load index decreased at‐site production of genetic migrants, while cti had a weak positive effect at‐site. The global model does appear to have support (Table 2), but the correlation structure of the parameters prevented convergence (producing a singularity error), making the AICc unreliable. In the global model, both connectivity of occupied sites (betweenness_ralu) and wetlands (centrality_wet) had parameter estimates with 95% confidence intervals not overlapping zero.

**TABLE 6:** Standardized parameter estimates and confidence intervals for the best supported gravity model (temperature + moisture) of functional connectivity for *Rana luteiventris* in the Jarbidge‐Independence region.

Parameter	Variable	Parameter estimate	Confidence interval
Distance	Distance	−0.106	0.016
At‐site	Heat load index	−0.312	0.127
	Compound topographic index	−0.038	0.039
Between‐site	Mean temperature of the warmest month	0.751	0.272
	Compound topographic index	0.008	0.003
	Autumn precipitation	−0.263	0.111

## 4 DISCUSSION

Genomic data can play an important role in delineating conservation units, informing our understanding of both neutral and adaptive differentiation in at‐risk species. However, these data will rarely be definitive on their own due in part to the difficulty of linking neutral differentiation with demographic independence (Palsbøll et al., 2007; Taylor & Dizon, 1999) and uncertainty associated with the identification of adaptive genetic variation (Hoban et al., 2016; Kardos & Shafer, 2018; Pearse, 2016). By integrating genomic inferences with complementary data, we can better inform CU delineation while acknowledging the uncertainty inherent in making decisions with imperfect and incomplete data (Funk et al., 2019). For Nevada *Rana luteiventris*, the integration of genomics with dispersal, site occupancy and landscape data allowed us to better evaluate CUs, while identifying areas of uncertainty.

### 4.1 Evolutionarily significant units within Nevada

The geographical isolation of the southernmost populations of *R. luteiventris* in Nevada has led to questions about their potential status as ESUs. The Ruby region is 80 km south of the Jarbidge‐Independence, with a lack of suitable intervening habitat or hydrological connectivity. The Toiyabe is even more distant, located ~320 km southeast of the Ruby Mountains.

Our genomic results clearly support reproductive isolation of the Ruby and Toiyabe, the first criterion for ESU status, based on neutral pairwise genetic divergence and population structure results. While population structure analyses do not identify these regions as the primary drivers of genetic differentiation within Nevada, current reproductive isolation is a certainty, given the lack of both intervening suitable habitat and hydrological connectivity. In support of mitochondrial results from a previous study (Funk et al., 2008), we found some evidence for shared genetic ancestry between Ruby and Maggie sites (in the Jarbidge‐Independence region, Figures 3, 4, 5) indicating that these populations were likely to have once been connected by gene flow.

While we did identify some signatures of adaptive differentiation across sites in the Jarbidge‐Independence, genomic data do not support adaptive divergence of the Ruby or Toiyabe, the second criterion for ESU delineation. While the adaptive analysis used here can be subject to false negatives (i.e., failure to detect an adaptive signature when it is present) and does not include all potential drivers of adaptive differentiation (e.g., for disease resistance), genetic diversity in these regions is extremely low, indicating that genetic drift is probably the dominant evolutionary force, potentially overpowering selection. While this does not necessarily preclude local adaptation, the current evidence indicates that these regions do not meet the threshold for adaptive differentiation that substantially contributes to the evolutionary legacy of the species.

However, even if these regions do not meet the threshold of ESU status, their disjunct locations at the southern range boundary are “significant to the species as a whole because…[their] loss would result in a substantial modification of the species' range” (Columbia Spotted Frog Technical Team, 2015). Restoring healthy, functional and self‐sustaining populations in these regions will therefore remain a priority for maintaining *R. luteiventris* throughout the historical Nevada range, one of the goals of the 2015 Conservation Agreement and Strategy plan. Importantly, because these regions do not represent areas of significant adaptive divergence based on these genomic results, management actions such as genetic rescue will remain viable options to increase genetic diversity in these isolated areas.

### 4.2 Management units

Management units are generally defined based on demographic independence (i.e., when dispersal rates are <10%; Moritz, 1999; Palsbøll et al., 2007). Translating genetic metrics of population differentiation into estimates of current rates of dispersal (the metric of interest for MUs) is challenging for a number of reasons, in particular because natural systems strongly deviate from many of the assumptions of population genetic models (e.g., Slatkin, 2005; Whitlock & McCauley, 1999). Additionally, methods for inferring contemporary dispersal from genetic data (e.g., Mussmann et al., 2019) require large sample sizes to detect migrants (*n* ≥ 30), a challenging prospect when funding is limited and/or there are many occupied sites distributed across complex landscapes. In the case of Nevada *R. luteiventris*, either approach would have been unrealistic to implement given the complex spatial structure among populations and variation in population sizes (clear violations of population genetic models), and the large number of occupied sites (*n* = 194).

To address these limitations, we combined our regionwide genetic sampling design with occupancy data that included 170 unsampled but occupied sites to project functional connectivity across the Jarbidge‐Independence. Projection of the model to include unsampled sites was essential because Nevada *R. luteiventris* show limited dispersal capacity, and restriction of the gravity models to the sampled locations would have provided little information on functional connectivity (e.g., only 6% of pairwise Euclidean distances among sampled genetic sites are below the maximum dispersal threshold of 15 km). While this approach does not provide a quantitative evaluation of demographic independence, our evaluation of functional connectivity was sufficient to meet the management goals for the species, which are not highly dependent on an assessment of demographic independence (Taylor & Dizon, 1999). Instead, the goal for MU delineation in Nevada *R. luteiventris* is to inform recovery efforts that will maintain genetic distinctiveness while maximizing the effectiveness of actions including genetic rescue and reintroductions (Columbia Spotted Frog Technical Team, 2015).

The use of watersheds as a basis for MUs in Nevada was based on previous work that identified ridgelines as barriers to movement for *R. luteiventris* in Idaho and Montana (Funk, Blouin, et al., 2005; Murphy et al., 2010). However, our connectivity results indicate that movement among Nevada populations may not be highly limited by watershed boundaries in some areas, especially in the core of the Jarbidge‐Independence region where ridgelines frequently represent only minor topographic features. Evidence for connectivity across watershed boundaries was particularly strong in the mid‐Bruneau (TEGU) and northern North Fork (NFHU). These sampling sites, including nearby unsampled sites that fall within the same fine‐scale watershed delineations (i.e., HUC10 or HUC12), could be combined with the larger Owyhee MU. Similarly, separation of the Independence Valley (TACA) into a separate MU was not supported by the genomic results, which assign this site primarily to the Willow Creek watershed. Despite model projection of functional connectivity with the North Fork basin, genomic results do not support connectivity between Independence Valley and North Fork sites. Connectivity projections indicate potential flow between the (unsampled) northern Independence Valley and southern South Fork Owyhee, though additional genotyping would be needed to verify these model projections.

Based on this integration of population structure and functional connectivity results, we suggest revision of the MU delineation for Nevada *R. luteiventris* (Figure 7; Figure S10). First, our results indicate that Merritt Creek and Winters Creek should be designated as new MUs due to their distinctiveness from the rest of the watershed unit. The sites included in the Merritt Creek unit had average pairwise *F*_ST_ values close to those of the geographically and hydrologically disjunct Ruby Mountains (0.648 for the Ruby Mountains and 0.635 for MERL and MERU). Similarly, the North Fork showed clear within‐watershed diversity, supporting an east–west split based on finer‐scale watershed delineations. Finally, the remainder of the large Bruneau River watershed MU was not assigned due to lack of data; in particular, the southern Bruneau sites have potential functional connectivity with Marys River, but additional genotyping would be needed to verify those projections.

Clearly, the Ruby and Toiyabe regions represent separate MUs, due to their geographical and hydrological isolation. However, genomic analyses do not support the division of the Toiyabe region into two MUs. While the average pairwise *F*_ST_ between the Big Smoky Valley site (FAPO) and Reese River sites is slightly higher (0.097) than the average within‐Reese River pairwise *F*_ST_ (0.071), the values are relatively low, especially considering that FAPO is at the southern range boundary. Additionally, PCA and admixture results do not clearly distinguish the FAPO site from others in the Toiyabe, despite little evidence for movement of marked frogs between the Big Smoky Valley and Reese River (D. S. Pilliod and others, unpublished data). These results indicate that the Toiyabe region could be managed as a single MU, especially when efforts are focused on improving genetic diversity across the region (next section).

### 4.3 Site evaluations and functional connectivity

The uniformly low effective sizes quantified in our study system are in line with estimates for other western ranids, including northern *R. luteiventris* populations (Phillipsen et al., 2011), though some of the Nevada sites clearly have extremely small effective sizes, even for amphibians. Because samples at each site represent frogs from different age classes, *N*_e_ estimates may be downwardly biased (Waples et al., 2014), and so represent conservative estimates. Sites with small *N*_e_ generally had reduced genetic diversity, reflecting the effects of genetic drift. By contrast, sites in the core part of the Jarbidge‐Independence range had higher estimates of both *N*_e_ and genetic diversity.

The levels of nucleotide diversity across all sites (mean = 0.0007, range = 0.00003–0.0017) were among the lowest found in sexually reproducing animals. For comparison, nucleotide diversity in endangered island foxes (*Urocyon littoralis*) of the California Channel Islands ranges from 0.00012 to 0.00082 (Funk et al., 2016). The Toiyabe region had uniformly low nucleotide diversity (all five sites = 0.00003), though *N*_e_ estimates varied. For example, the southernmost sampled site, FAPO, had the lowest *N*_e_ estimate (3.0, range 2.1–11.6), while two sites in the Reese River had higher estimates (point estimates of 44.7 and 49.0). Increasing *N*_e_ at some locations probably reflects the efficacy of ongoing habitat enhancement efforts in the Toiyabe that began in 2004, including drought mitigation efforts (i.e., constructing and enlarging breeding ponds) and the use of grazing exclosures (Pilliod et al., 2021). Long‐term population monitoring in the Toiyabe (2005–2018) showed substantial increases in census population sizes, from a population estimate of 582 in 2005 to 1957 in 2017 (with a peak estimate of 3560 in 2012; USFWS, 2015a; Columbia Spotted Frog Technical Team, 2018). This pattern of low diversity in populations with relatively large census sizes has been found in other aquatic species subject to periodic drought conditions in the Great Basin, including Toiyabe populations of the threatened Lahontan cutthroat trout (*Oncorhynchus clarkii henshawi*) (Peacock & Dochtermann, 2012).

Despite small *N*_e_ and overall low genetic diversity, we identified signatures of adaptive differentiation in the eastern part of the core range, with Salmon Falls sites showing adaptive divergence related to relatively high summer precipitation, and Marys River sites related to low winter temperatures. The Winters Creek site in South Fork Owyhee showed a weaker adaptive signature related to high autumn precipitation and higher winter minimum temperatures. These findings are preliminary without further validation; however, the limited adaptive divergence found across the range indicates that the risk of outbreeding depression across MUs should be minimal during management activities such as genetic rescue (Fitzpatrick et al., 2016; Frankham et al., 2011).

Functional connectivity was mediated by temperature and moisture, with higher temperatures reducing at‐site productivity and between‐site connectivity and wetter conditions increasing at‐site productivity and between‐site connectivity. This agrees with a recent analysis of 220 sites across the Great Basin, which reported that *R. luteiventris* occupancy increased with increasing precipitation and decreased with increasing temperature at the watershed scale (Smith & Goldberg, 2020). Support for the importance of connectivity of occupied sites and wetlands in the global model suggests that spatial arrangement in relation to other wetlands, particularly those that have been occupied, may contribute to functional connectivity. Additionally, connectivity was reduced as the distance between sites increased (IBD), illustrating the importance of maintaining networks of suitable habitat to facilitate gene flow in desert landscapes. A previous study that included Great Basin *R. luteiventris* in southeast Oregon and Idaho similarly found steep IBD relationships in those desert landscapes and functional connectivity driven by temperature–moisture constraints (Robertson et al., 2018). In contrast, studies of higher latitude populations of *R. luteiventris* in mountain environments have identified ridgelines and elevation as restricting gene flow (Funk, Blouin, et al., 2005; Murphy et al., 2010) and higher temperatures as increasing at‐site productivity (Murphy et al., 2010). These contrasting results reflect how diverse ecological settings alter the landscape template for functional connectivity and productivity, even within a species range (Bull et al., 2011; Robertson et al., 2018).

A core goal of the most recent Conservation Agreement and Strategy plan was to identify important corridors for movement among sites in Nevada (Columbia Spotted Frog Technical Team, 2015). Flow predictions from the top gravity model highlight potential areas of higher connectivity, including terrestrial upland habitats. An important caveat of these predictions is that they represent a straight‐line sample of the landscape between sites based on the selected model, rather than a specific movement path. For example, there are several occupied sites in the Owyhee drainage that act as central nodes for many high‐flow edges. These sites and their surrounding habitats are good candidates for additional field investigation to identify and, where possible, mitigate impediments to movement for *R. luteiventris* in this stronghold of genetic diversity for Nevada populations. This also provides an opportunity to test connectivity models developed using circuit theory, including those projected into the future under different climate scenarios (Pilliod et al., 2015). Predicted flow across the South Fork Owyhee and Independence basins, as well as across the Bruneau and Marys basins represent hypotheses to be investigated in the field (e.g., through habitat evaluation and occupancy searches) as well as in any follow‐up genetic work.

### 4.4 Summary of conservation recommendations for Nevada *R. luteiventris*

The Jarbidge‐Independence region represents the only known reservoir of relatively high genetic variation remaining for Nevada populations. Management actions that maintain a large, functional network of populations and high‐quality habitat across this core region could reduce risks to the DPS from stochastic events and environmental fluctuations, including extended drought periods. This will also help maintain genetic diversity and evolutionary potential, which will be important for translocation and genetic rescue efforts to peripheral, genetically depauperate populations.

Due to their smaller *N*_e_, lower genetic diversity and isolation, peripheral populations are at much higher risk of reduced fitness due to inbreeding depression and loss of evolutionary potential (Forester et al., in press; Gilpin & Soulé, 1986). These populations are also at high risk from ecological processes such as demographic stochasticity, environmental extremes (particularly long‐duration drought events; Pilliod et al., 2021), invasive predatory species and disease (Arkle & Pilliod, 2015). The most serious risks to *R. luteiventris* populations in peripheral areas differ depending on the basin, but two main concerns are bullfrog invasion and habitat loss/degradation due to anthropogenic alterations to regional hydrology and climate change. In locations where bullfrogs are thought to be causing population declines and extirpations, managers could consider translocations to bullfrog‐free habitats as an effective, primary option. Where possible, translocations that include genetic rescue could also enhance genetic diversity and evolutionary potential (Bell et al., 2019; Ralls et al., 2018, 2020), work that is currently in progress in the western part of the Jarbidge‐Independence based on these results. In regions where maintaining genetic distinctiveness is important, selecting source populations based on genetic similarity (e.g., from PCA, admixture and predicted flow results) and/or geographical proximity would be helpful, even if those populations have reduced genetic variability. However, where maximizing genetic rescue and fitness effects is most important, selecting sites with higher diversity (i.e., from the core of the Jarbidge‐Independence) would be best, especially because risks of outbreeding depression should be low (Bell et al., 2019; Frankham, 2015).

Where habitat loss and degradation are the driving factors in population declines, improvements in habitat quality that restore natural hydrology can clearly boost population sizes, as shown by restoration efforts in the Toiyabe region and elsewhere (Pilliod et al., 2021; Pilliod & Scherer, 2015). Climate change in the Great Basin is already increasing winter temperatures and aridity, and expanding the frequency and duration of droughts (Snyder et al., 2019). Restoration of resilient hydrological regimes will be incredibly difficult, especially for snow‐dominated regimes, but creative solutions may be necessary to support recovery of *R. luteiventris* and other threatened and sensitive species in these desert systems. Finally, while habitat restoration in peripheral sites can increase census population sizes, it will not restore genetic diversity in the absence of genetic rescue efforts, as discussed above.

### 4.5 General recommendations

Our study highlights both the strengths and the limitations of genomic data in informing diverse management questions in complex natural systems, and how integration of genomic with complementary data can improve downstream inferences. First, by carefully designing our genetic sampling across the Nevada range, we were able to identify unexpected patterns of population connectivity across watershed boundaries that highlight the uniqueness of desert populations of this widely distributed species. This genetic sampling design required the unavoidable trade‐off between sampling more sites and genotyping more individuals per site. In our case, we sacrificed sample size at any given site to improve geographical and environmental coverage across the range, a design that allowed us to better meet the diverse management goals of the study. Despite careful planning, we still have areas of uncertainty that will require follow‐up if further resolution is needed (e.g., unassigned units in Figure 7). Transparency about uncertainty and the limitations of sampling designs and analytical inferences is an essential component of both effective communication with management partners and application of study results to conservation actions.

Second, we took advantage of complementary data that included capture–recapture and range‐wide survey data to improve our inference of functional connectivity and MU delineation in the absence of information on demographic connectivity. A dispersal threshold derived from capture–recapture data allowed us to apply a biologically relevant limit on the connectivity model, improving its relevance for delineating MUs and highlighting movement corridors that are more likely to be ecologically relevant. Similarly, site occupancy data allowed us to predict the gravity model beyond the small subset of genomic sampling sites, expanding our capacity to infer connectivity across the landscape. This approach provides a framework for MU delineation in other species where funding limitations either prevent genotyping of all occupied sites or do not allow for sufficient sample sizes to evaluate contemporary dispersal using genomic analyses. More generally, communication with managers on the functional definition of an MU that best meets the species management goals is critical to designing an effective and beneficial conservation genomics study.

Finally, genomic‐scale data allowed us to identify adaptive differentiation, or in this case, the relative lack of it, which is useful for informing ESU delineation and genetic rescue efforts. A lack of genomic resources in many at‐risk species, such as high‐quality, annotated reference genomes, combined with the practical difficulties of experimental approaches for demonstrating local adaptation in fragile, declining populations (especially in cases where generation times are long) will continue to hamper the utility of genomic assessments of evolutionary potential in at‐risk species (Funk et al., 2019). Despite these limitations, anonymous genomic assessments of candidate adaptive differentiation, such as the approach used here, remain useful in identifying spatial patterns of differentiation to inform CU delineation as well as reducing the risk of potential outbreeding depression during genetic rescue. Overall, while a single genetic data set is unlikely to be able to fully address all potential conservation and management questions for a given species, a carefully planned study, designed in collaboration with practitioners to address priority management questions, can provide diverse insights for conservation and recovery actions.

## AUTHOR CONTRIBUTIONS

B.R.F. and W.C.F. designed the research in consultation with C.M. and J.P. C.M., J.P., R.V.H. and J.H. collected and coordinated the collection of tissue samples; D.S.P. provided capture–recapture data. B.R.F. and M.M. performed the research and analysed the data. B.R.F. wrote the paper; all authors edited and provided feedback on subsequent versions.

## CONFLICT OF INTEREST

The authors declare that they have no conflict of interest.

## BENEFIT SHARING STATEMENT

Benefits from this research accrue from the sharing of our data and results on public databases as described above, contributing to the conservation of biodiversity.

## Supporting information


Figures S1–S10
Table S1–S5

## Data Availability

Raw, demultiplexed sequencing data are available on the NCBI Sequence Read Archive under BioProject PRJNA869693: https://www.ncbi.nlm.nih.gov/bioproject/PRJNA869693. Filtered VCFs and metadata are available on Dryad (Forester et al., 2022): https://doi.org/10.5061/dryad.w6m905qqn.

## References

[mec16660-bib-0001] Akaike, H. (1973). Information theory and an extension of the maximum likelihood principle. In B. Petrov & F. Csaki (Eds.), Second international symposium on information theory (pp. 267–281). Akademia Kiado.

[mec16660-bib-0002] Alexander, D. H. , Novembre, J. , & Lange, K. (2009). Fast model‐based estimation of ancestry in unrelated individuals. Genome Research, 19, 1655–1664. 10.1101/gr.094052.109 19648217 PMC2752134

[mec16660-bib-0003] Allendorf, F. W. , Hohenlohe, P. A. , & Luikart, G. (2010). Genomics and the future of conservation genetics. Nature Reviews Genetics, 11(10), 697–709. 10.1038/nrg2844 20847747

[mec16660-bib-0004] Anderson, E. C. (2019). whoa: Evaluation of genotyping error in genotype‐by‐sequencing data (R package version 0.0.1) . https://CRAN.R‐project.org/package=whoa

[mec16660-bib-0005] Andrews, S. (2019). *FastQC: A quality control tool for high throughput sequence data* (Version 0.11.8). http://www.bioinformatics.babraham.ac.uk/projects/fastqc/

[mec16660-bib-0006] Arkle, R. S. , & Pilliod, D. S. (2015). Persistence at distributional edges: Columbia spotted frog habitat in the arid Great Basin, USA. Ecology and Evolution, 5(17), 3704–3724. 10.1002/ece3.1627 26380699 PMC4567874

[mec16660-bib-0007] Bell, D. A. , Robinson, Z. L. , Funk, W. C. , Fitzpatrick, S. W. , Allendorf, F. W. , Tallmon, D. A. , & Whiteley, A. R. (2019). The exciting potential and remaining uncertainties of genetic rescue. Trends in Ecology & Evolution, 34(12), 1070–1079. 10.1016/j.tree.2019.06.006 31296345

[mec16660-bib-0008] Bonacich, P. , & Lloyd, P. (2001). Eigenvector‐like measures of centrality for asymmetric relations. Social Networks, 23(3), 191–201. 10.1016/S0378-8733(01)00038-7

[mec16660-bib-0009] Brandes, U. (2001). A faster algorithm for betweenness centrality. The Journal of Mathematical Sociology, 25(2), 163–177. 10.1080/0022250X.2001.9990249

[mec16660-bib-0010] Bull, R. A. , Cushman, S. A. , Mace, R. , Chilton, T. , Kendall, K. C. , Landguth, E. L. , Schwartz, M. K. , McKelvey, K. , Allendorf, F. W. , & Luikart, G. (2011). Why replication is important in landscape genetics: American black bear in the Rocky Mountains. Molecular Ecology, 20(6), 1092–1107. 10.1111/j.1365-294X.2010.04944.x 21261764

[mec16660-bib-0011] Burnham, K. , & Anderson, D. (2002). Model selection and multimodel inference (2nd ed.). Springer‐Verlag.

[mec16660-bib-0012] Capblancq, T. , & Forester, B. R. (2021). Redundancy analysis: A Swiss Army Knife for landscape genomics. Methods in Ecology and Evolution, 12, 2298–2309. 10.1111/2041-210X.13722

[mec16660-bib-0013] Cayuela, H. , Dorant, Y. , Forester, B. R. , Jeffries, D. L. , Mccaffery, R. M. , Eby, L. A. , Hossack, B. R. , JMW, G. , Pilliod, D. S. , & Chris Funk, W. (2021). Genomic signatures of thermal adaptation are associated with clinal shifts of life history in a broadly distributed frog. Journal of Animal Ecology, 91, 1222–1238. 10.1111/1365-2656.13545 34048026 PMC9292533

[mec16660-bib-0014] Chang, C. C. , Chow, C. C. , Tellier, L. C. , Vattikuti, S. , Purcell, S. M. , & Lee, J. J. (2015). Second‐generation PLINK: Rising to the challenge of larger and richer datasets. GigaScience, 4(1), 7. 10.1186/s13742-015-0047-8 25722852 PMC4342193

[mec16660-bib-0015] Columbia Spotted Frog Technical Team . (2003a). Conservation agreement and conservation strategy: Columbia spotted frog (Rana luteiventris) Toiyabe, Great Basin subpopulation, Nevada. Columbia Spotted Frog Technical Team.

[mec16660-bib-0016] Columbia Spotted Frog Technical Team . (2003b). Conservation agreement and strategy: Columbia spotted frog (Rana luteiventris) Great Basin population, Nevada, Northeastern subpopulations; Jarbidge‐Independence and Ruby Mountain. Columbia Spotted Frog Technical Team.

[mec16660-bib-0017] Columbia Spotted Frog Technical Team . (2015). Conservation Agreement and Conservation Strategy for Columbia Spotted Frogs (Rana luteiventris) in Nevada. Columbia Spotted Frog Technical Team.

[mec16660-bib-0018] Columbia Spotted Frog Technical Team . (2018). Columbia Spotted Frog (Rana luteiventris), Toiyabe Subpopulation Conservation Agreement and Strategy Implementation, 2017‐2018 Annual Report. Columbia Spotted Frog Technical Team.

[mec16660-bib-0019] Csardi, G. , & Nepusz, T. (2006). The igraph software package for complex network research. InterJournal, Complex Systems, 1695, 1–9. https://igraph.org

[mec16660-bib-0020] Daly, C. , Halbleib, M. , Smith, J. I. , Gibson, W. P. , Doggett, M. K. , Taylor, G. H. , Curtis, J. , & Pasteris, P. P. (2008). Physiographically sensitive mapping of climatological temperature and precipitation across the conterminous United States. International Journal of Climatology, 28(15), 2031–2064. 10.1002/joc.1688

[mec16660-bib-0021] De Reu, J. , Bourgeois, J. , Bats, M. , Zwertvaegher, A. , Gelorini, V. , De Smedt, P. , Chu, W. , Antrop, M. , De Maeyer, P. , Finke, P. , Van Meirvenne, M. , Verniers, J. , & Crombé, P. (2013). Application of the topographic position index to heterogeneous landscapes. Geomorphology, 186, 39–49. 10.1016/j.geomorph.2012.12.015

[mec16660-bib-0022] Dewitz, J. (2020). National Land Cover Database (NLCD) 2016 Products. U.S. Geological Survey data release . 10.5066/P96HHBIE

[mec16660-bib-0023] Diestel, R. (2005). Graph theory (3rd ed.). Springer‐Verlag.

[mec16660-bib-0024] Do, C. , Waples, R. S. , Peel, D. , Macbeth, G. M. , Tillett, B. J. , & Ovenden, J. R. (2014). NeEstimator v2: Re‐implementation of software for the estimation of contemporary effective population size (Ne) from genetic data. Molecular Ecology Resources, 14(1), 209–214. 10.1111/1755-0998.12157 23992227

[mec16660-bib-0025] Duan, N. (1983). Smearing estimate: A nonparametric retransformation method. Journal of the American Statistical Association, 78(383), 605–610. 10.2307/2288126

[mec16660-bib-0026] Evans, J. S. (2021). SpatialEco (R package version 1.3‐6) . https://github.com/jeffreyevans/spatialEco

[mec16660-bib-0027] Evans, J. S , & Murphy, M. A. (2020). GeNetIt (R package version 0.1‐3) . https://github.com/jeffreyevans/GeNetIt

[mec16660-bib-0028] Fitzpatrick, S. W. , Gerberich, J. C. , Angeloni, L. M. , Bailey, L. L. , Broder, E. D. , Torres‐Dowdall, J. , Handelsman, C. A. , López‐Sepulcre, A. , Reznick, D. N. , Ghalambor, C. K. , & Funk, W. C. (2016). Gene flow from an adaptively divergent source causes rescue through genetic and demographic factors in two wild populations of Trinidadian guppies. Evolutionary Applications, 9(7), 879–891. 10.1111/eva.12356 27468306 PMC4947150

[mec16660-bib-0029] Forester, B. R. , Beever, E. A. , Darst, C. , Szymanski, J. , & Funk, W. C. (2022). Linking evolutionary potential to extinction risk: Applications and future directions. Frontiers in Ecology and the Environment, 1–9. 10.1002/fee.2552

[mec16660-bib-0030] Forester, B. R. , Lasky, J. R. , Wagner, H. H. , & Urban, D. L. (2018). Comparing methods for detecting multilocus adaptation with multivariate genotype–environment associations. Molecular Ecology, 27, 2215–2233. 10.1111/mec.14584 29633402

[mec16660-bib-0031] Forester, B. R. , Murphy, M. , Mellison, C. , Petersen, J. , Pilliod, D. S. , Van Horne, R. , Harvey, J. , & Funk, W. C. (2022). Data from: Genomics‐informed delineation of conservation units in a desert amphibian. Dryad, Dataset, 10.5061/dryad.w6m905qqn PMC9804278 35976166

[mec16660-bib-0032] Fortuna, M. A. , Gómez‐Rodríguez, C. , & Bascompte, J. (2006). Spatial network structure and amphibian persistence in stochastic environments. Proceedings of the Royal Society B: Biological Sciences, 273(1592), 1429–1434. 10.1098/rspb.2005.3448 PMC1560303 16777733

[mec16660-bib-0033] Fotheringham, A. , & O'Kelly, M. (1989). Spatial interaction models: Formulation and applications. Kluwer Academic.

[mec16660-bib-0034] Frankham, R. (2015). Genetic rescue of small inbred populations: Meta‐analysis reveals large and consistent benefits of gene flow. Molecular Ecology, 24(11), 2610–2618. 10.1111/mec.13139 25740414

[mec16660-bib-0035] Frankham, R. , Ballou, J. D. , Eldridge, M. D. B. , Lacy, R. C. , Ralls, K. , Dudash, M. R. , & Fenster, C. B. (2011). Predicting the probability of outbreeding depression. Conservation Biology, 25(3), 465–475. 10.1111/j.1523-1739.2011.01662.x 21486369

[mec16660-bib-0036] Fraser, D. J. , & Bernatchez, L. (2001). Adaptive evolutionary conservation: Towards a unified concept for defining conservation units. Molecular Ecology, 10(12), 2741–2752. 10.1046/j.0962-1083.2001.01411.x 11903888

[mec16660-bib-0037] Freeman, L. C. (1978). Centrality in social networks: Conceptual clarification. Social Networks, 1(3), 215–239. 10.1016/0378-8733(78)90021-7

[mec16660-bib-0038] Frichot, E. , & François, O. (2015). LEA: An R package for landscape and ecological association studies. Methods in Ecology and Evolution, 6(8), 925–929. 10.1111/2041-210X.12382

[mec16660-bib-0039] Funk, W. C. , Blouin, M. S. , Corn, P. S. , Maxell, B. A. , Pilliod, D. S. , Amish, S. , & Allendorf, F. W. (2005). Population structure of Columbia spotted frogs (*Rana luteiventris*) is strongly affected by the landscape. Molecular Ecology, 14(2), 483–496. 10.1111/j.1365-294X.2005.02426.x 15660939

[mec16660-bib-0040] Funk, W. C. , Forester, B. R. , Converse, S. J. , Darst, C. , & Morey, S. (2019). Improving conservation policy with genomics: A guide to integrating adaptive potential into U.S. Endangered Species Act decisions for conservation practitioners and geneticists. Conservation Genetics, 20(1), 115–134. 10.1007/s10592-018-1096-1

[mec16660-bib-0041] Funk, W. C. , Greene, A. E. , Corn, P. S. , & Allendorf, F. W. (2005). High dispersal in a frog species suggests that it is vulnerable to habitat fragmentation. Biology Letters, 1(1), 13–16. 10.1098/rsbl.2004.0270 17148116 PMC1629065

[mec16660-bib-0111] Funk, W. C. , Lovich, R. E. , Hohenlohe, P. A. , Hofman, C. A. , Morrison, S. A. , Sillett, T. S. , Ghalambor, C. K. , Maldonado, J. E. , Rick, T. C. , Day, M. D. , Polato, N. R. , Fitzpatrick, S. W. , Coonan, T. J. , Crooks, K. R. , Dillon, A. , Garcelon, D. K. , King, J. L. , Boser, C. L. , Gould, ,N. , … Andelt, W. F. (2016). Adaptive divergence despite strong genetic drift: Genomic analysis of the evolutionary mechanisms causing genetic differentiation in the island fox (*Urocyon littoralis*). Molecular Ecology, 25(10), 2176–2194. 10.1111/mec.13605 26992010 PMC4877267

[mec16660-bib-0042] Funk, W. C. , Pearl, C. A. , Draheim, H. M. , Adams, M. J. , Mullins, T. D. , & Haig, S. M. (2008). Range‐wide phylogeographic analysis of the spotted frog complex (*Rana luteiventris* and *Rana pretiosa*) in northwestern North America. Molecular Phylogenetics and Evolution, 49(1), 198–210. 10.1016/j.ympev.2008.05.037 18606551

[mec16660-bib-0043] Gilpin, M. , & Soulé, M. (1986). Minimum viable populations: Processes of species extinctions. In M. Soulé (Ed.), Conservation biology: The science of scarcity and diversity (pp. 13–34). Sinauer Associates.

[mec16660-bib-0044] Gosselin, T. , Lamothe, M. , Devloo‐Delva, F. , & Grewe, P. (2020). radiator: RADseq Data Exploration, Manipulation and Visualization using R (R package version 1.1.6) . https://thierrygosselin.github.io/radiator/

[mec16660-bib-0045] Gugger, P. F. , Liang, C. T. , Sork, V. L. , Hodgskiss, P. , & Wright, J. W. (2018). Applying landscape genomic tools to forest management and restoration of Hawaiian koa (*Acacia koa*) in a changing environment. Evolutionary Applications, 11(2), 231–242. 10.1111/eva.12534 29387158 PMC5775490

[mec16660-bib-0046] Gustafson, R. G. , Waples, R. S. , Myers, J. M. , Weitkamp, L. A. , Bryant, G. J. , Johnson, O. W. , & Hard, J. J. (2007). Pacific salmon extinctions: Quantifying lost and remaining diversity. Conservation Biology, 21(4), 1009–1020. 10.1111/j.1523-1739.2007.00693.x 17650251

[mec16660-bib-0047] Haertel, J. D. , Owczarak, A. , & Storm, R. M. (1974). A comparative study of the chromosomes from five species of the genus *Rana* (Amphibia: Salientia). Copeia, 1974(1), 109–114.

[mec16660-bib-0048] Hesselbarth, M. H. K. , Sciaini, M. , With, K. A. , Wiegand, K. , & Nowosad, J. (2019). landscapemetrics: An open‐source R tool to calculate landscape metrics. Ecography, 42(10), 1648–1657. 10.1111/ecog.04617

[mec16660-bib-0049] Hoban, S. , Kelley, J. L. , Lotterhos, K. E. , Antolin, M. F. , Bradburd, G. , Lowry, D. B. , Poss, M. L. , Reed, L. K. , Storfer, A. , & Whitlock, M. C. (2016). Finding the genomic basis of local adaptation: Pitfalls, practical solutions, and future directions. The American Naturalist, 188(4), 379–397. 10.1086/688018 PMC5457800 27622873

[mec16660-bib-0050] Jombart, T. , & Ahmed, I. (2011). adegenet 1.3‐1: New tools for the analysis of genome‐wide SNP data. Bioinformatics (Oxford, England), 27(21), 3070–3071. 10.1093/bioinformatics/btr521 21926124 PMC3198581

[mec16660-bib-0051] Kamvar, Z. N. , Brooks, J. C. , & Grünwald, N. J. (2015). Novel R tools for analysis of genome‐wide population genetic data with emphasis on clonality. Frontiers in Genetics, 6, 208. 10.3389/fgene.2015.00208 26113860 PMC4462096

[mec16660-bib-0052] Kardos, M. , & Shafer, A. B. A. (2018). The peril of gene‐targeted conservation. Trends in Ecology & Evolution, 33(11), 827–839. 10.1016/j.tree.2018.08.011 30241778

[mec16660-bib-0053] Krueger, F. (2019). *Trim Galore*! (Version 0.6.4). http://www.bioinformatics.babraham.ac.uk/projects/trim_galore/

[mec16660-bib-0054] Lowe, W. H. , & Allendorf, F. W. (2010). What can genetics tell us about population connectivity? Molecular Ecology, 19(15), 3038–3051. 10.1111/j.1365-294X.2010.04688.x 20618697

[mec16660-bib-0055] Martin, M. (2011). Cutadapt removes adapter sequences from high‐throughput sequencing reads. EMBnet.Journal, 17(1), 10–12. 10.14806/ej.17.1.200

[mec16660-bib-0056] McAdoo, K. , & Mellison, C . (2017). Case study: Successful collaboration for Columbia spotted frog conservation in Northern and Central Nevada . Fact Sheet 16‐10, University of Nevada Cooperative Extension, Reno, Nevada.

[mec16660-bib-0057] McCune, B. , & Keon, D. (2002). Equations for potential annual direct incident radiation and heat load. Journal of Vegetation Science, 13(4), 603–606. 10.1111/j.1654-1103.2002.tb02087.x

[mec16660-bib-0058] Mills, L. S. , & Allendorf, F. W. (1996). The one‐migrant‐per‐generation rule in conservation and management. Conservation Biology, 10(6), 1509–1518. 10.1046/j.1523-1739.1996.10061509.x

[mec16660-bib-0059] Mims, M. C. , Moore, C. E. , & Shadle, E. J. (2020). Threats to aquatic taxa in an arid landscape: Knowledge gaps and areas of understanding for amphibians of the American Southwest. WIREs Water, 7(4), e1449. 10.1002/wat2.1449

[mec16660-bib-0060] Moore, I. , Gessler, P. , Nielsen, G. , & Petersen, G. (1993). Terrain attributes and estimation methods and scale effects. In A. Jakeman , M. Beck , & M. McAleer (Eds.), Modeling Change in Environmental Systems (pp. 189–214). Wiley.

[mec16660-bib-0061] Moritz, C. (1999). Conservation units and translocations: Strategies for conserving evolutionary processes. Hereditas, 130(3), 217–228. 10.1111/j.1601-5223.1999.00217.x

[mec16660-bib-0062] Munger, J. C. , Gerber, M. , Madrid, K. , Carroll, M.‐A. , Petersen, W. , & Heberger, L. (1998). U.S. National Wetland Inventory classifications as predictors of the occurrence of Columbia spotted frogs (*Rana luteiventris*) and Pacific treefrogs (*Hyla regilla*). Conservation Biology, 12(2), 320–330.

[mec16660-bib-0063] Murphy, M. A. , Dezzani, R. , Pilliod, D. S. , & Storfer, A. (2010). Landscape genetics of high mountain frog metapopulations. Molecular Ecology, 19(17), 3634–3649. 10.1111/j.1365-294X.2010.04723.x 20723055

[mec16660-bib-0064] Mussmann, S. M. , Douglas, M. R. , Chafin, T. K. , & Douglas, M. E. (2019). BA3‐SNPs: Contemporary migration reconfigured in BayesAss for next‐generation sequence data. Methods in Ecology and Evolution, 10(10), 1808–1813. 10.1111/2041-210X.13252

[mec16660-bib-0065] Naujokaitis‐Lewis, I. R. , Rico, Y. , Lovell, J. , Fortin, M.‐J. , & Murphy, M. A. (2013). Implications of incomplete networks on estimation of landscape genetic connectivity. Conservation Genetics, 14(2), 287–298. 10.1007/s10592-012-0385-3

[mec16660-bib-0066] Oksanen, J. , Simpson, G. L. , Blanchet, F. G. , Kindt, R. , Legendre, P. , Minchin, P. R. , O'Hara, R. B. , Solymos, P. , Stevens, M. H. H. , Szoecs, E. , Wagner, H. , Barbour, M. , Bedward, M. , Bolker, B. , Borcard, D. , Carvalho, G. , Chirico, M. , De Caceres, M. , Durand, S. , … Weedon, J. (2019). vegan: Community Ecology Package (R package version 2.5‐6) . http://CRAN.R‐project.org/package=vegan

[mec16660-bib-0067] Palsbøll, P. J. , Bérubé, M. , & Allendorf, F. W. (2007). Identification of management units using population genetic data. Trends in Ecology & Evolution, 22(1), 11–16. 10.1016/j.tree.2006.09.003 16982114

[mec16660-bib-0068] Paris, J. R. , Stevens, J. R. , & Catchen, J. M. (2017). Lost in parameter space: A road map for stacks. Methods in Ecology and Evolution, 8, 1360–1373. 10.1111/2041-210X.12775

[mec16660-bib-0069] Peacock, M. M. , & Dochtermann, N. A. (2012). Evolutionary potential but not extinction risk of Lahontan cutthroat trout (*Oncorhynchus clarkii henshawi*) is associated with stream characteristics. Canadian Journal of Fisheries and Aquatic Sciences, 69(4), 615–626. 10.1139/f2012-006

[mec16660-bib-0070] Pearse, D. E. (2016). Saving the spandrels? Adaptive genomic variation in conservation and fisheries management. Journal of Fish Biology, 89(6), 2697–2716. 10.1111/jfb.13168 27723095

[mec16660-bib-0071] Pembleton, L. W. , Cogan, N. O. I. , & Forster, J. W. (2013). StAMPP: An R package for calculation of genetic differentiation and structure of mixed‐ploidy level populations. Molecular Ecology Resources, 13(5), 946–952. 10.1111/1755-0998.12129 23738873

[mec16660-bib-0072] Peterson, B. K. , Weber, J. N. , Kay, E. H. , Fisher, H. S. , & Hoekstra, H. E. (2012). Double digest RADseq: An inexpensive method for de novo SNP discovery and genotyping in model and non‐model species. PLoS ONE, 7(5), e37135. 10.1371/journal.pone.0037135 22675423 PMC3365034

[mec16660-bib-0073] Phillipsen, I. C. , Funk, W. C. , Hoffman, E. A. , Monsen, K. J. , & Blouin, M. S. (2011). Comparative analyses of effective population size within and among species: Ranid frogs as a case study. Evolution, 65(10), 2927–2945. 10.1111/j.1558-5646.2011.01356.x 21967433

[mec16660-bib-0074] Pike, R. , & Wilson, S. (1971). Elevation‐relief ratio, hypsometric integral, and geomorphic area‐altitude analysis. GSA Bulletin, 82(4), 1079–1084. 10.1130/0016-7606(1971)82[1079:ERHIAG]2.0.CO;2

[mec16660-bib-0075] Pilliod, D. S. , Arkle, R. S. , Robertson, J. M. , Murphy, M. A. , & Funk, W. C. (2015). Effects of changing climate on aquatic habitat and connectivity for remnant populations of a wide‐ranging frog species in an arid landscape. Ecology and Evolution, 5(18), 3979–3994. 10.1002/ece3.1634 26445654 PMC4588645

[mec16660-bib-0076] Pilliod, D. S. , Hausner, M. B. , & Scherer, R. D. (2021). From satellites to frogs: Quantifying ecohydrological change, drought mitigation, and population demography in desert meadows. Science of The Total Environment, 758, 143632. 10.1016/j.scitotenv.2020.143632 33218818

[mec16660-bib-0077] Pilliod, D. S. , & Scherer, R. D. (2015). Managing habitat to slow or reverse population declines of the Columbia spotted frog in the Northern Great Basin. The Journal of Wildlife Management, 79(4), 579–590. 10.1002/jwmg.868

[mec16660-bib-0078] R Core Team . (2020). R: A language and environment for statistical computing. R Foundation for Statistical Computing http://www.R‐project.org

[mec16660-bib-0079] Ralls, K. , Ballou, J. D. , Dudash, M. R. , Eldridge, M. D. B. , Fenster, C. B. , Lacy, R. C. , Sunnucks, P. , & Frankham, R. (2018). Call for a paradigm shift in the genetic management of fragmented populations. Conservation Letters, 11(2), e12412. 10.1111/conl.12412

[mec16660-bib-0080] Ralls, K. , Sunnucks, P. , Lacy, R. C. , & Frankham, R. (2020). Genetic rescue: A critique of the evidence supports maximizing genetic diversity rather than minimizing the introduction of putatively harmful genetic variation. Biological Conservation, 251, 108784. 10.1016/j.biocon.2020.108784

[mec16660-bib-0081] Reaser, J. (1996). *Rana pretiosa* (spotted frog): Vagility. Herpetological Review, 27, 196–197.

[mec16660-bib-0082] Rehfeldt, G. (2006). *A spline model of climate for the Western United States* (No. General Technical Report RMRS‐GTR‐165; p. 21). U.S. Department of Agriculture, Forest Service, Rocky Mountain Research Station.

[mec16660-bib-0083] Robertson, J. M. , Murphy, M. A. , Pearl, C. A. , Adams, M. J. , Páez‐Vacas, M. I. , Haig, S. M. , Pilliod, D. S. , Storfer, A. , & Funk, W. C. (2018). Regional variation in drivers of connectivity for two frog species (*Rana pretiosa* and *R. luteiventris*) from the U.S. Pacific Northwest. Molecular Ecology, 27(16), 3242–3256. 10.1111/mec.14798 30010212

[mec16660-bib-0084] Rochette, N. C. , Rivera‐Colón, A. G. , & Catchen, J. M. (2019). Stacks 2: Analytical methods for paired‐end sequencing improve RADseq‐based population genomics. Molecular Ecology, 28(21), 4737–4754. 10.1111/mec.15253 31550391

[mec16660-bib-0085] Saito, L. , Byer, S. , Badik, K. , McGwire, K. , Provencher, L. , & Minor, B. (2020). Mapping indicators of groundwater dependent ecosystems in Nevada: Important resources for a water‐limited state. Journal of the Nevada Water Resources Association Winter 2020, 1, 48–72. 10.22542/jnwra/2020/1/3

[mec16660-bib-0086] Schweyen, H. , Rozenberg, A. , & Leese, F. (2014). Detection and removal of PCR duplicates in population genomic ddRAD studies by addition of a Degenerate Base Region (DBR) in sequencing adapters. The Biological Bulletin, 227(2), 146–160. 10.1086/BBLv227n2p146 25411373

[mec16660-bib-0087] Slatkin, M. (2005). Seeing ghosts: The effect of unsampled populations on migration rates estimated for sampled populations. Molecular Ecology, 14(1), 67–73. 10.1111/j.1365-294X.2004.02393.x 15643951

[mec16660-bib-0088] Smith, M. M. , & Goldberg, C. S. (2020). Occupancy in dynamic systems: Accounting for multiple scales and false positives using environmental DNA to inform monitoring. Ecography, 43(3), 376–386. 10.1111/ecog.04743

[mec16660-bib-0089] Snyder, K. A. , Evers, L. , Chambers, J. C. , Dunham, J. , Bradford, J. B. , & Loik, M. E. (2019). Effects of changing climate on the hydrological cycle in cold desert ecosystems of the Great Basin and Columbia Plateau. Rangeland Ecology & Management, 72(1), 1–12. 10.1016/j.rama.2018.07.007

[mec16660-bib-0090] Taylor, B. L. , & Dizon, A. E. (1999). First policy then science: Why a management unit based solely on genetic criteria cannot work. Molecular Ecology, 8(s1), S11–S16. 10.1046/j.1365-294X.1999.00797.x 10703548

[mec16660-bib-0091] Turner, R. (2021). *deldir: Delaunay Triangulation and Dirichlet (Voronoi) Tessellation* (R package version 0.2‐10). https://CRAN.R‐project.org/package=deldir

[mec16660-bib-0092] USFWS & NMFS [U.S. Fish and Wildlife Service & National Marine Fisheries Service] . (1996). Policy regarding the recognition of distinct vertebrate population segments under the Endangered Species Act. Federal Register, 61, 4722–4725.

[mec16660-bib-0093] USFWS [U.S. Fish and Wildlife Service] . (1993). Endangered and threatened wildlife and plants; Finding on petition to list the Spotted frog. Federal Register, 58, 27262–27263.

[mec16660-bib-0094] USFWS [U.S. Fish and Wildlife Service] . (2015a). Endangered and threatened wildlife and plants; 12‐month findings on petitions to list 19 species as endangered or threatened species. Federal Register, 80(195), 60834–60850.

[mec16660-bib-0095] USFWS [U.S. Fish and Wildlife Service] . (2015b). Species status assessment report for the Columbia spotted frog (Rana luteiventris), Great Basin distinct population segment. Reno Fish and Wildlife Office.

[mec16660-bib-0110] USGS [U.S. Geological Survey] Gap Analysis Project . (2018). Columbia Spotted Frog (Rana luteiventris) aCOFRx_CONUS_2001v1 Range Map. U.S. Geological Survey data release. 10.5066/F7NV9HCP

[mec16660-bib-0109] Waples, R. S. (1991). Pacific salmon, Oncorhynchus spp., and the definition of “species” under the Endangered Species Act. Marine Fisheries Review, 53(3), 11–22.

[mec16660-bib-0096] Waples, R. K. , Larson, W. A. , & Waples, R. S. (2016). Estimating contemporary effective population size in non‐model species using linkage disequilibrium across thousands of loci. Heredity, 117(4), 233–240. 10.1038/hdy.2016.60 27553452 PMC5026758

[mec16660-bib-0097] Waples, R. S. (2006). Distinct population segments. In J. M. Scott , D. Goble , & F. Davis (Eds.), The Endangered Species Act at thirty, Volume 2: Conserving biodiversity in human‐dominated landscapes (pp. 127–149). Island Press.

[mec16660-bib-0098] Waples, R. S. , & Anderson, E. C. (2017). Purging putative siblings from population genetic data sets: A cautionary view. Molecular Ecology, 26(5), 1211–1224. 10.1111/mec.14022 28099771

[mec16660-bib-0099] Waples, R. S. , Antao, T. , & Luikart, G. (2014). Effects of overlapping generations on linkage disequilibrium estimates of effective population size. Genetics, 197(2), 769–780. 10.1534/genetics.114.164822 24717176 PMC4063931

[mec16660-bib-0100] Waples, R. S. , & Lindley, S. T. (2018). Genomics and conservation units: The genetic basis of adult migration timing in Pacific salmonids. Evolutionary Applications, 11(9), 1518–1526. 10.1111/eva.12687 30344624 PMC6183503

[mec16660-bib-0101] Watts, A. G. , Schlichting, P. E. , Billerman, S. M. , Jesmer, B. R. , Micheletti, S. , Fortin, M. J. , Funk, W. C. , Hapeman, P. , Muths, E. , & Murphy, M. A. (2015). How spatio‐temporal habitat connectivity affects amphibian genetic structure. Frontiers in Genetics, 6, 275. 10.3389/fgene.2015.00275 26442094 PMC4561841

[mec16660-bib-0102] Weckworth, B. V. , Hebblewhite, M. , Mariani, S. , & Musiani, M. (2018). Lines on a map: Conservation units, meta‐population dynamics, and recovery of woodland caribou in Canada. Ecosphere, 9(7), e02323. 10.1002/ecs2.2323

[mec16660-bib-0103] Weeks, A. R. , Stoklosa, J. , & Hoffmann, A. A. (2016). Conservation of genetic uniqueness of populations may increase extinction likelihood of endangered species: The case of Australian mammals. Frontiers in Zoology, 13(1), 31. 10.1186/s12983-016-0163-z 27398088 PMC4939060

[mec16660-bib-0104] Weir, B. S. , & Cockerham, C. C. (1984). Estimating F‐statistics for the analysis of population structure. Evolution, 38(6), 1358–1370. 10.2307/2408641 28563791

[mec16660-bib-0105] Welch, N. E. , & MacMahon, J. A. (2005). Identifying habitat variables important to the rare Columbia Spotted Frog in Utah (U.S.A.): An information‐theoretic approach. Conservation Biology, 19(2), 473–481. 10.1111/j.1523-1739.2005.00384.x

[mec16660-bib-0106] Whiteley, A. R. , Fitzpatrick, S. W. , Funk, W. C. , & Tallmon, D. A. (2015). Genetic rescue to the rescue. Trends in Ecology & Evolution, 30(1), 42–49. 10.1016/j.tree.2014.10.009 25435267

[mec16660-bib-0107] Whitlock, Ms. C. , & McCauley, D. E. (1999). Indirect measures of gene flow and migration: Fst≠1/(4Nm+1). Heredity, 82(2), 117–125. 10.1038/sj.hdy.6884960 10098262

[mec16660-bib-0108] Zuur, A. , Ieno, E. , Walker, N. , Saveliev, A. , & Smith, G. (2009). Mixed effects models and extensions in ecology with R. Springer‐Verlag.

