# Supplementary material for: Genomics‐informed delineation of conservation units in a desert amphibian
Source: Mol Ecol. 2022 Aug 30;31(20):5249–69. doi: 10.1111/mec.16660 (PMC9804278; doi:10.1111/mec.16660)
Supplement: Supplementary file 1 — Figures S1–S10 Table S1–S5 [file MEC-31-5249-s001.docx]

**Supplemental Information for:**

**Genomics-informed delineation of**

**conservation units in a desert amphibian**

Brenna R. Forester, Melanie Murphy, Chad Mellison, Jeffrey Petersen,

David S. Pilliod, Rachel Van Horne, Jim Harvey, W. Chris Funk


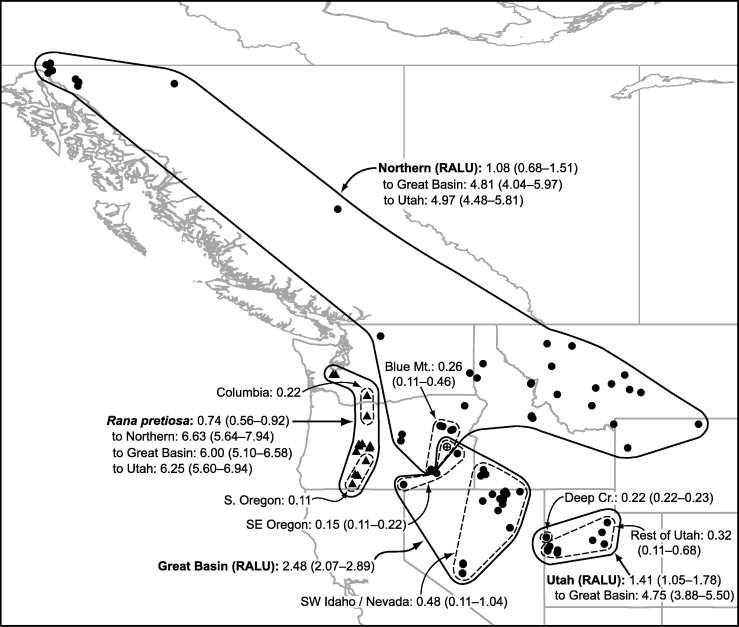


**Figure S1:** Geographic distribution of major clades (solid black lines) and nested clades (dashed lines) identified in the mitochondrial DNA phylogenetic analyses of Funk et al. 2008, with mean percent corrected sequence divergence (and ranges in parentheses) shown within and among clades. Note that this figure shows both *Rana pretiosa* and *R. luteiventris* (RALU) clades. Figure reproduced from Funk et al. 2008.


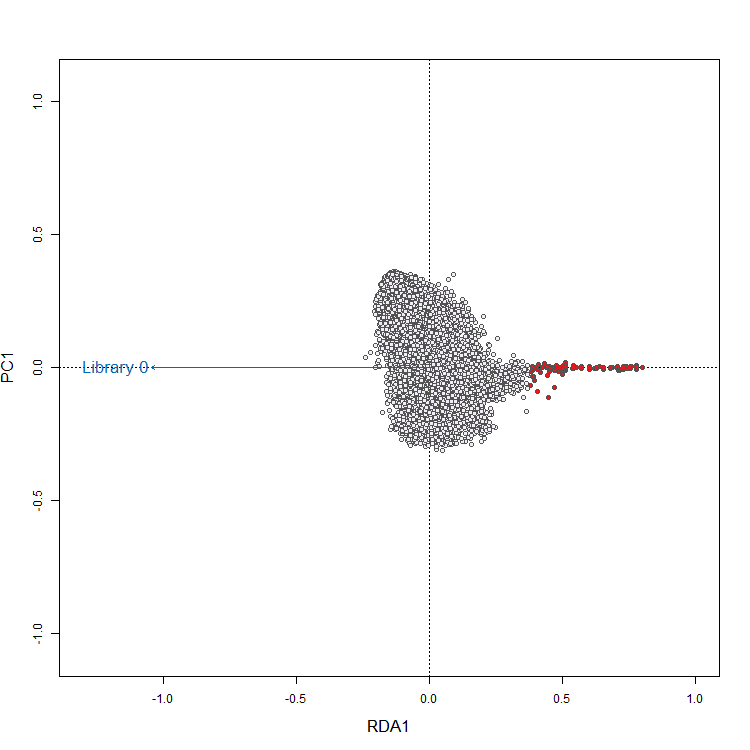


**Figure S2**. Redundancy analysis plot of 40,390 SNPs using dummy coded library number as a predictor (0 for problematic library, 1 for all other libraries). SNPs identified as contributing to the lane effect (476 total) are shown in red (+/- 4 standard deviations from the mean loading on RDA axis 1).


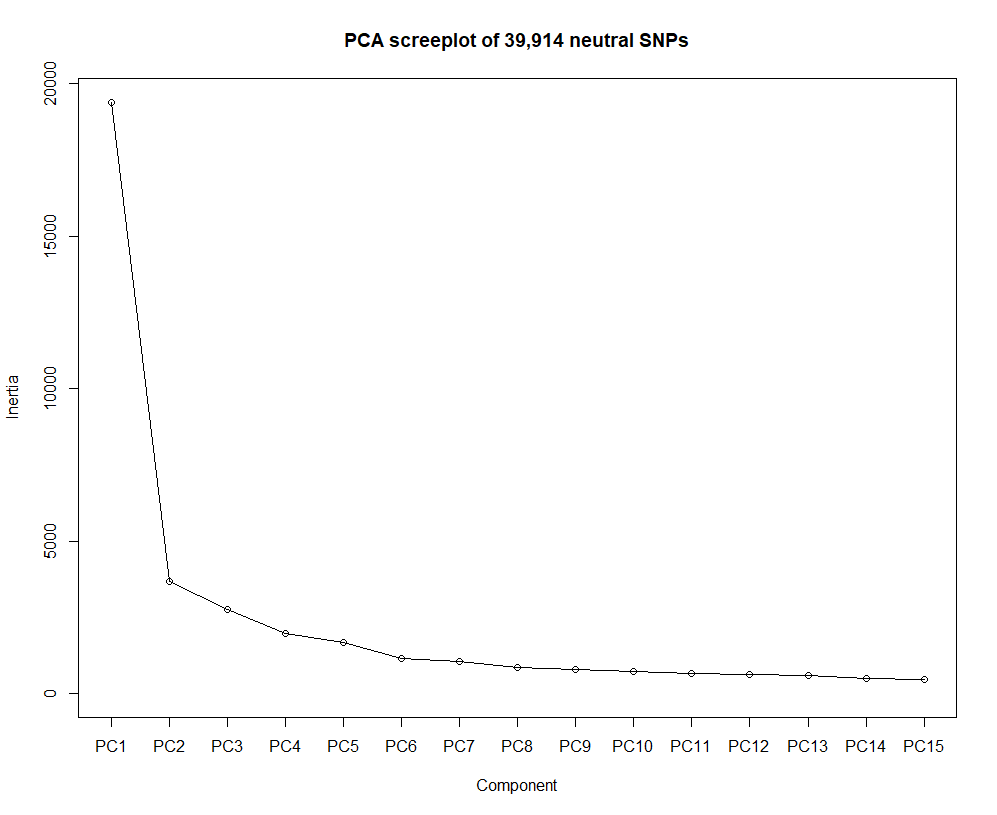


**Figure S3**. Screeplot of PCA eigenvalues (aka “Inertia”) from analysis of site-based data for 39,914 neutral SNPs.

**
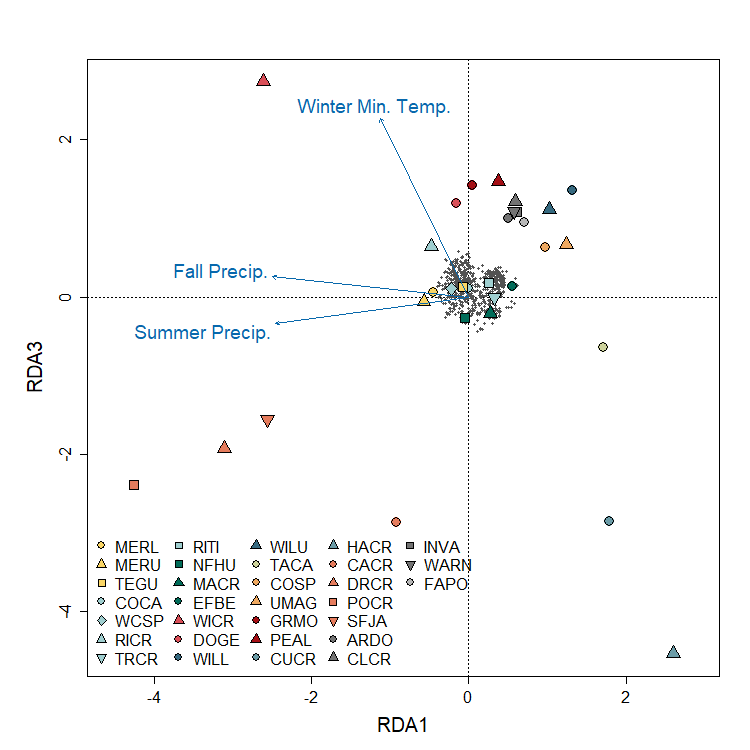

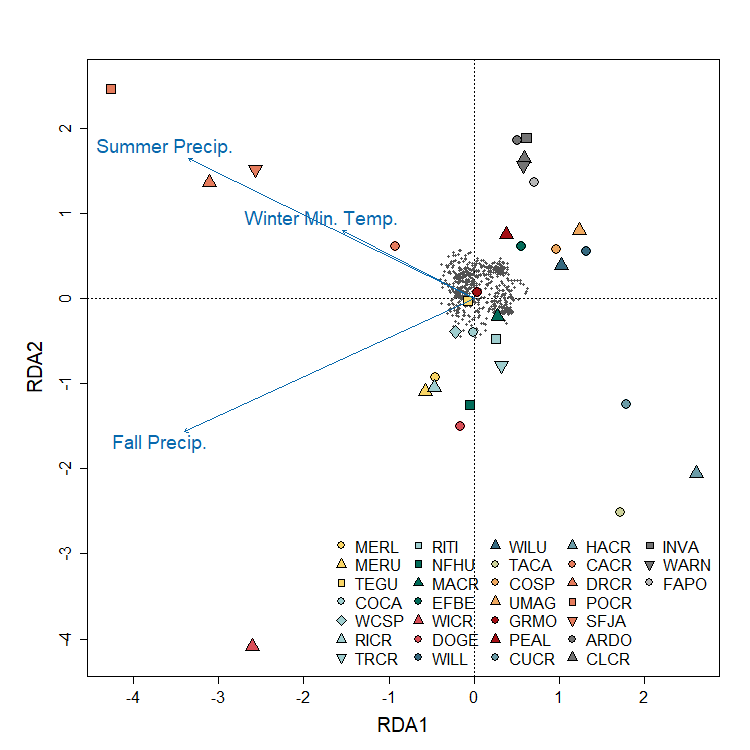
**

**Figure S4**. Site-based RDA of 689 candidate adaptive markers; RDA axes 1 and 2 at left and axes 1 and 3 at right.


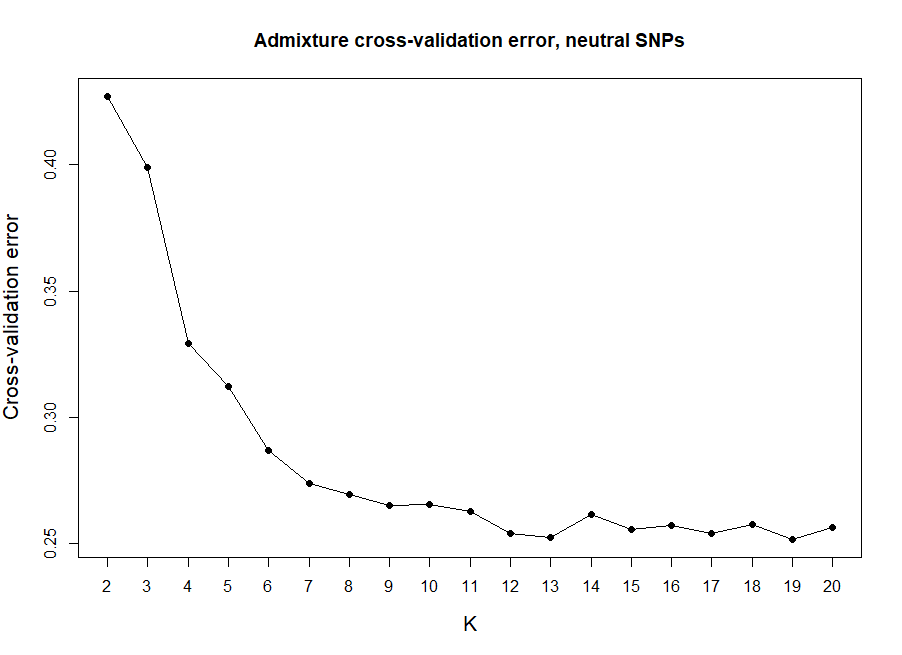


**Figure S5**. Admixture cross-validation errors for K values from 2 to 20.


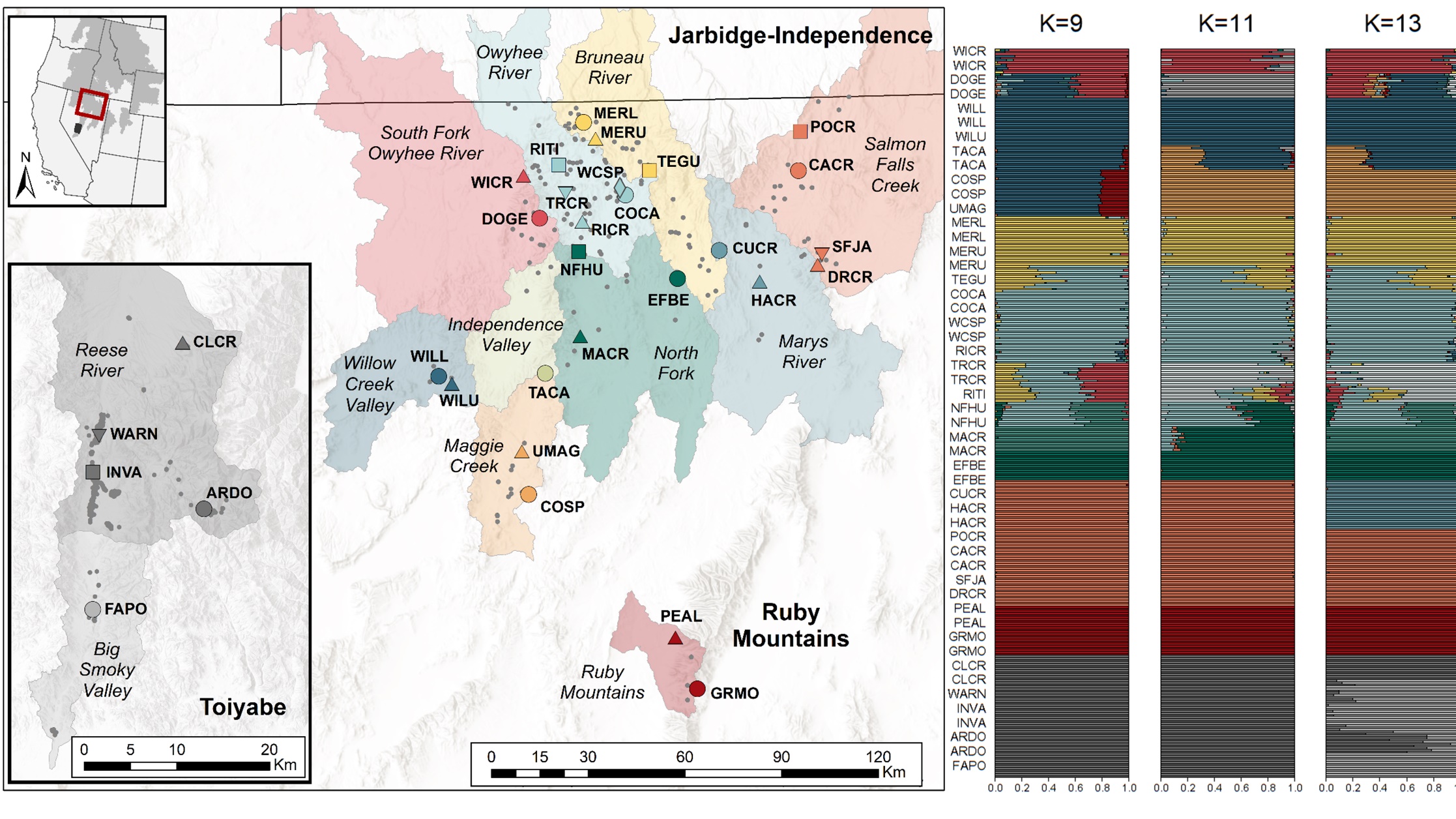
**Figure S6**: Sampling map and Admixture assignment plots for K=9, 11, and 13 using 39,225 neutral SNPs in 357 individuals distributed across 31 sampling sites. Admixture colors match primary watersheds on the map, except for light green in K=9 and 13, white in K=11 and 13, and light grey in K=11, which indicate genetic differentiation not related to watershed delineation. While sample order of the Admixture plots has been reorganized for this visualization, the plots show identical results to Fig. 5 (main text).


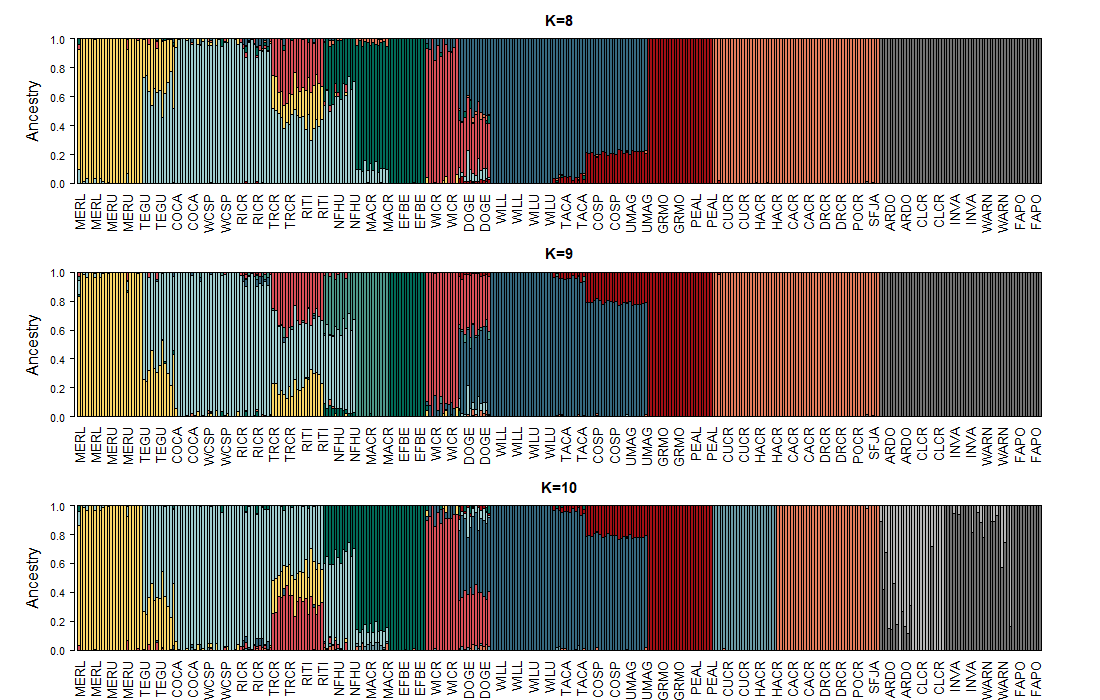
**Figure S7**: Admixture assignment plots for K=8, 9, and 10 using 39,225 neutral SNPs in 357 individuals distributed across 31 sampling sites. Colors match primary watersheds in Fig. 1, except for light green in K=9, which indicates genetic differentiation not related to watershed delineation.


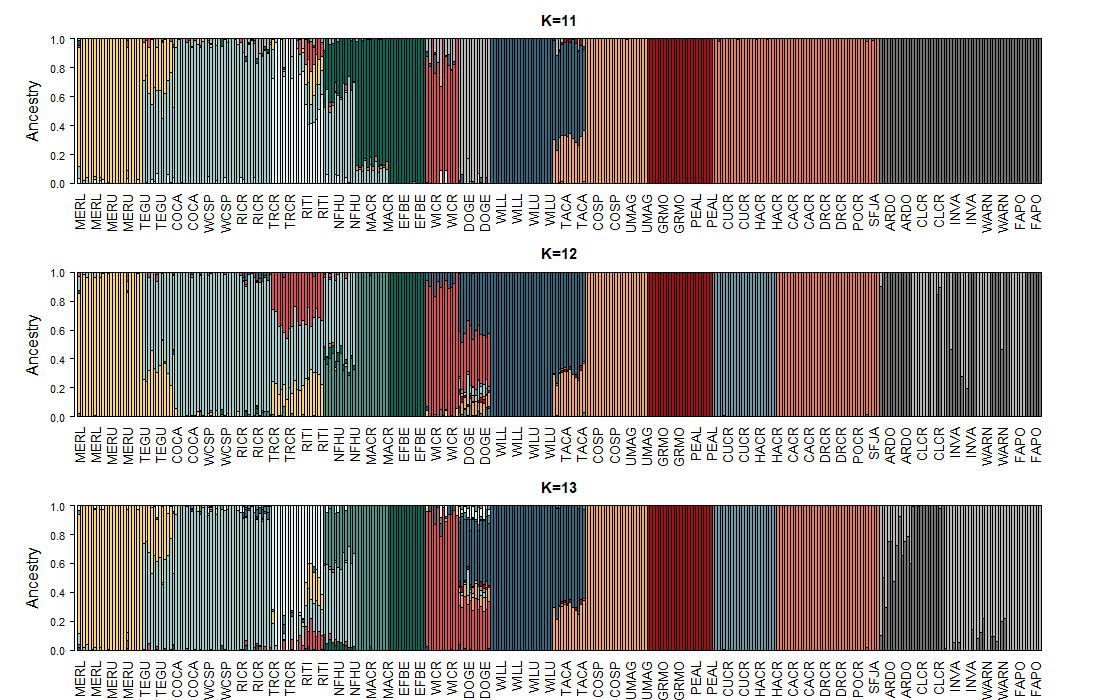
**Figure S8**: Admixture assignment plots for K=11, 12, and 13 using 39,225 neutral SNPs in 357 individuals distributed across 31 sampling sites. Colors match primary watersheds in Fig. 1, except for light green in K=12 and 13, white in K=11 and 13, and light grey in K=11, which indicate genetic differentiation not related to watershed delineation.


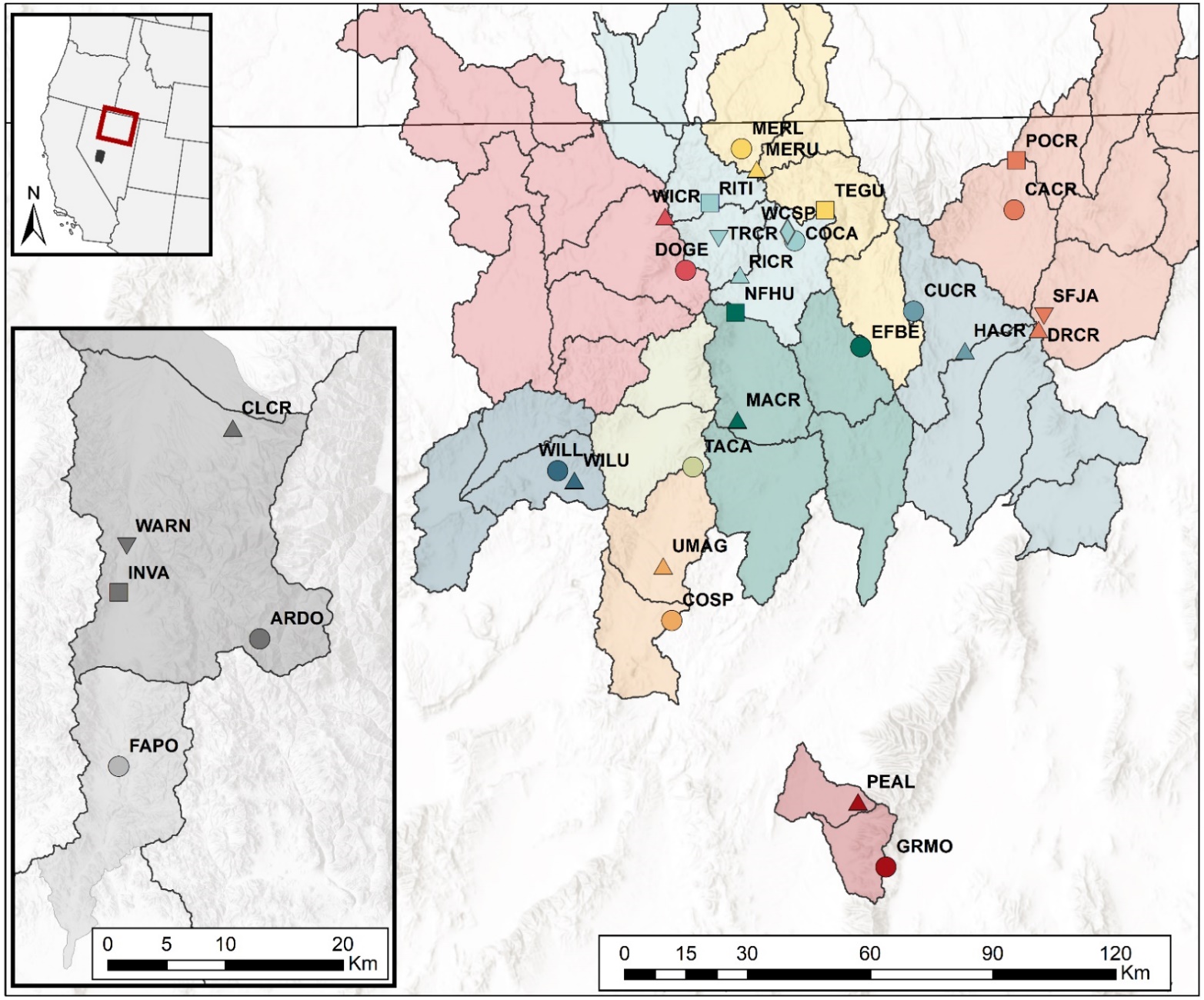


**Figure S9**: Locations of 31 *R. luteiventris* sites sampled for genomic data in Nevada (large shapes with four letter labels). Sampling locations are color coded based on watershed/current management unit; gray lines represent HUC10 watershed units.


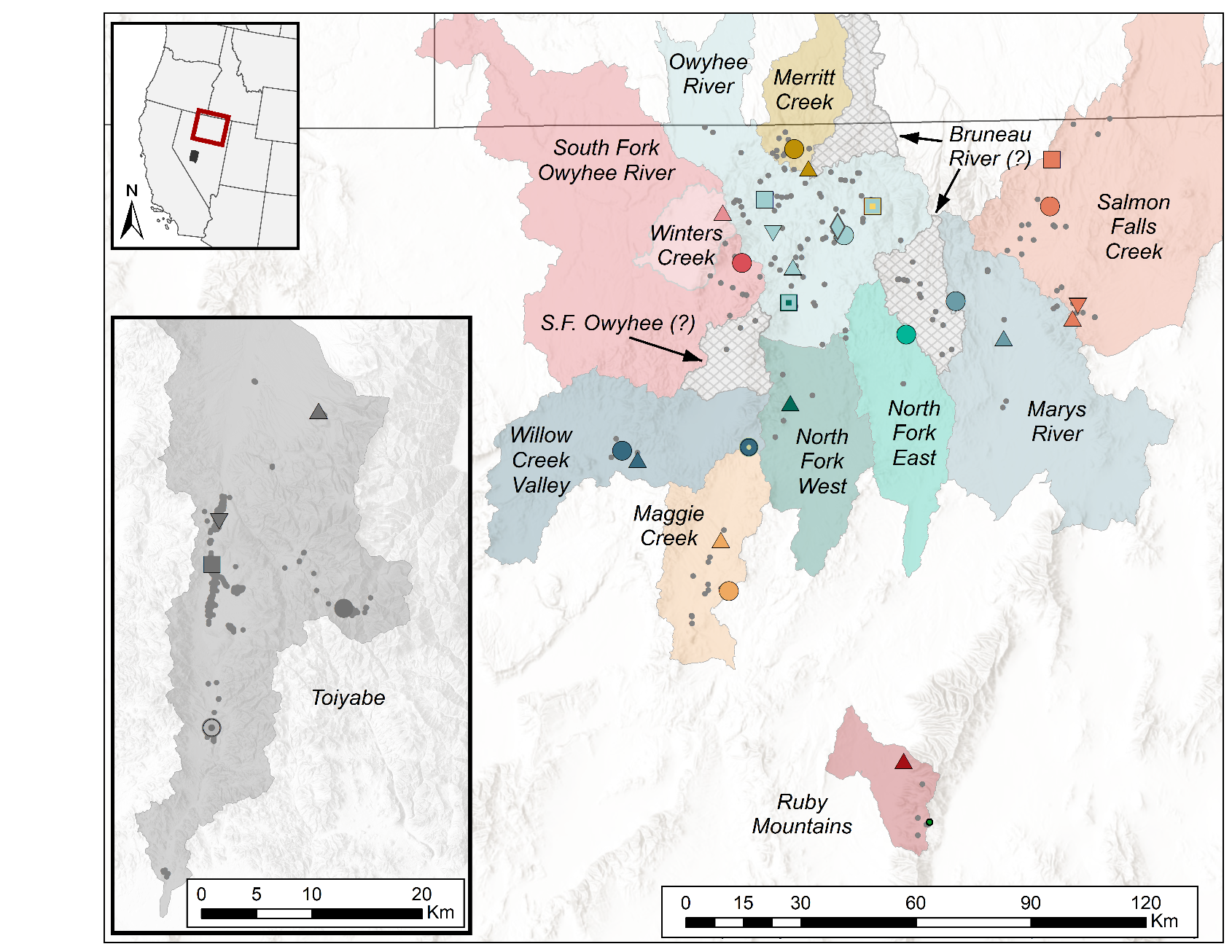


**Figure S10**: Possible reassignment of management units for *R. luteiventris* populations in Nevada. Suggested MUs are labeled in italics, with unassigned units shown in grey with diamond hatching. Genomic site colors/shapes follow Fig. 1; dual-color squares and circles represent genomic sites that were reassigned to a new/different MU.

**Table S1**: Metadata for genetic sampling sites.

| Site name | Site code | Management Unit | Final # individuals | Elevation (m) | Easting | Northing | Summer precip (mm) | Fall precip (mm) | Winter min. temp (°C) |
| --- | --- | --- | --- | --- | --- | --- | --- | --- | --- |
| Lower Merritt Creek | MERL | Bruneau | 12 | 1912 | 592251 | 4643092 | 19.4 | 38.5 | -8.5 |
| Upper Merritt Creek | MERU |  | 12 | 2169 | 595887 | 4638312 | 23.7 | 49.7 | -8.4 |
| Tennessee Gulch | TEGU |  | 12 | 2253 | 612721 | 4628322 | 24.5 | 44.1 | -9.1 |
| Coleman Canyon | COCA | Owyhee | 12 | 2045 | 605258 | 4620604 | 19.4 | 33.4 | -9.0 |
| Riffle Creek | RICR |  | 12 | 2153 | 591893 | 4612338 | 21.7 | 48.0 | -8.4 |
| Rio Tinto Ponds | RITI |  | 7 | 1793 | 584504 | 4629921 | 16.2 | 31.1 | -9.3 |
| Trail Creek | TRCR |  | 12 | 1775 | 586685 | 4621169 | 14.9 | 25.7 | -10.1 |
| Warm Creek Stock Pond | WCSP |  | 12 | 2009 | 603569 | 4622872 | 19.0 | 32.9 | -9.3 |
| Doby George Creek | DOGE | South Fork Owyhee | 12 | 1959 | 578630 | 4613412 | 18.0 | 38.6 | -8.7 |
| Winters Creek | WICR |  | 12 | 2217 | 573614 | 4626538 | 23.6 | 62.2 | -7.7 |
| Canyon Creek | CACR | Salmon Falls | 12 | 1833 | 658923 | 4628181 | 19.6 | 26.9 | -11.1 |
| Dry Creek | DRCR |  | 12 | 2218 | 664855 | 4598949 | 28.0 | 48.8 | -8.7 |
| Pole Creek | POCR |  | 8 | 2238 | 659485 | 4640193 | 31.7 | 48.8 | -8.8 |
| South Fork Jake Creek | SFJA |  | 6 | 2209 | 666190 | 4602221 | 24.6 | 42.0 | -8.8 |
| Cutt Creek | CUCR | Marys | 12 | 1927 | 634357 | 4603427 | 17.7 | 30.3 | -9.5 |
| Hanks Creek | HACR |  | 12 | 1759 | 646872 | 4593786 | 15.4 | 21.2 | -11.1 |
| East Fork Beaver | EFBE | North Fork | 14 | 1907 | 621444 | 4594715 | 15.7 | 26.0 | -10.5 |
| Mahala Creek | MACR |  | 12 | 1970 | 591246 | 4576835 | 15.9 | 28.1 | -9.6 |
| North Fork Humboldt River | NFHU |  | 12 | 2024 | 590775 | 4603022 | 21.5 | 47.1 | -10.3 |
| Tayler Canyon | TACA | Independence | 12 | 1931 | 580395 | 4565372 | 12.9 | 29.8 | -10.5 |
| Cottonwood Spring | COSP | Maggie | 12 | 1752 | 575205 | 4527827 | 13.4 | 23.9 | -9.1 |
| Upper Maggie Creek | UMAG |  | 11 | 1679 | 573111 | 4541148 | 13.2 | 23.7 | -9.7 |
| Lower Willow Creek | WILL | Willow | 12 | 1702 | 547336 | 4564434 | 12.8 | 25.4 | -8.7 |
| Upper Willow Creek | WILU |  | 11 | 1763 | 551397 | 4562091 | 13.1 | 25.5 | -8.8 |
| Site Name | **Site Code** | **MU** | **Final # individuals** | **Elevation (m)** | **Easting** | **Northing** | **Summer precip (mm)** | **Fall precip (mm)** | **Winter min. temp (°C)** |
| Green Mountain Creek | GRMO | Ruby | 12 | 2381 | 627741 | 4467650 | 19.4 | 43.1 | -7.7 |
| Pete Aleyo Pond | PEAL |  | 12 | 2194 | 620763 | 4483523 | 18.0 | 36.4 | -8.2 |
| Arc Dome | ARDO | Reese | 12 | 2471 | 468334 | 4292010 | 29.5 | 34.8 | -9.6 |
| Clear Creek | CLCR |  | 12 | 2333 | 466038 | 4309927 | 23.2 | 25.1 | -7.3 |
| Indian Valley | INVA |  | 12 | 2230 | 456347 | 4295989 | 23.3 | 23.2 | -8.7 |
| Upper Warners | WARN |  | 12 | 2181 | 457019 | 4299896 | 21.9 | 20.9 | -8.8 |
| Farrington Pond | FAPO | Big Smoky | 12 | 2083 | 456338 | 4281162 | 19.2 | 19.1 | -8.3 |

**Table S2**: Heterozygote miscall rate (posterior mean estimate of the heterozygote miscall rate), 95% confidence intervals (CIs), mean read depth, number of samples, and number of SNPs for each genetic sampling site.

| Site | Mean heterozygote miscall rate | Low 95% CI | High 95% CI | Mean read depth | Number of samples | Number of SNPs |
| --- | --- | --- | --- | --- | --- | --- |
| ARDO | 0.0008 | 0.000003 | 0.0030 | 27.26 | 12 | 940 |
| CACR | 0.0007 | 0.000001 | 0.0040 | 33.95 | 12 | 5575 |
| CLCR | 0.0012 | 0.00001 | 0.0042 | 27.72 | 12 | 1231 |
| COCA | 0.0230 | 0.0207 | 0.0250 | 26.68 | 12 | 25166 |
| COSP | 0.0002 | 0.000001 | 0.0006 | 30.87 | 12 | 5102 |
| CUCR | 0.0286 | 0.0223 | 0.0341 | 35.56 | 12 | 6855 |
| DOGE | 0.0216 | 0.0188 | 0.0264 | 31.49 | 12 | 26831 |
| DRCR | 0.0001 | 0.000001 | 0.0005 | 30.61 | 12 | 3282 |
| EFBE | 0.0001 | 0.0000004 | 0.0003 | 23.82 | 14 | 13546 |
| FAPO | 0.0238 | 0.0081 | 0.0452 | 37.92 | 12 | 934 |
| GRMO | 0.0003 | 0.000003 | 0.0014 | 31.35 | 12 | 3499 |
| HACR | 0.0002 | 0.000001 | 0.0007 | 32.24 | 12 | 6748 |
| INPA | 0.0007 | 0.000002 | 0.0034 | 29.71 | 12 | 1009 |
| MACR | 0.0001 | 0.000001 | 0.0002 | 33.39 | 12 | 20732 |
| MERL | 0.0104 | 0.0078 | 0.0131 | 32.58 | 12 | 25499 |
| MERU | 0.0001 | 0.0000003 | 0.0002 | 34.13 | 12 | 24108 |
| NFHU | 0.0195 | 0.0166 | 0.0221 | 30.69 | 12 | 28259 |
| PEAL | 0.0455 | 0.0371 | 0.0536 | 28.00 | 12 | 2636 |
| RICR | 0.0219 | 0.0196 | 0.0245 | 31.53 | 12 | 27351 |
| TACA | 0.0013 | 0.00001 | 0.0027 | 30.55 | 12 | 10151 |
| TEGU | 0.0485 | 0.0455 | 0.0511 | 26.79 | 12 | 27105 |
| TRCR | 0.00004 | 0.0000001 | 0.0001 | 26.56 | 12 | 30101 |
| UMAG | 0.0297 | 0.0225 | 0.0382 | 21.15 | 11 | 6648 |
| WARN | 0.0009 | 0.000004 | 0.0033 | 32.57 | 12 | 807 |
| WCSP | 0.0026 | 0.0006 | 0.0054 | 29.98 | 12 | 27248 |
| WICR | 0.0001 | 0.0000004 | 0.0002 | 32.08 | 12 | 29759 |
| WILL | 0.0002 | 0.000001 | 0.0005 | 23.46 | 12 | 6048 |
| WILU | 0.0063 | 0.0027 | 0.0098 | 28.75 | 11 | 7151 |
|  |  |  |  |  |  |  |
| Minimum | 0.00004 | 0.00000 | 0.00013 | 21.15 | 11 | 807 |
| Mean | 0.01030 | 0.00794 | 0.01335 | 30.05 | 12 | 13369 |
| Median | 0.00104 | 0.00000 | 0.00368 | 30.65 | 12 | 7003 |
| Maximum | 0.04846 | 0.04550 | 0.05356 | 37.92 | 14 | 30101 |

**Table S3**: Pairwise Fst values, organized by Management Unit. Values are scaled from highest (red) to lowest (blue). All Fst p-values

are < 0.0001 except INVA and WARN, which had a p-value of 0.037.

**Table S4**. AMOVA results for different Management Unit delineations.

| Groups | No. of groups | Variance components | % of variation |
| --- | --- | --- | --- |
| Current (watershed-based) MUs | 12 | Among groups | 61.0 |
|  |  | Among sites | 12.2 |
|  |  | Within sites | 26.8 |
| HUC2 | 2 | Among groups | 22.9 |
|  |  | Among sites | 52.2 |
|  |  | Within sites | 24.8 |
| HUC4 / HUC6 | 4 | Among groups | 34.2 |
|  |  | Among sites | 41.4 |
|  |  | Within sites | 24.4 |
| HUC8 | 10 | Among groups | 58.4 |
|  |  | Among sites | 14.9 |
|  |  | Within sites | 26.6 |
| HUC10 | 19 | Among groups | 65.8 |
|  |  | Among sites | 6.9 |
|  |  | Within sites | 27.3 |
| admixture7 | 7 | Among groups | 61.3 |
|  |  | Among sites | 13.5 |
|  |  | Within sites | 25.1 |
| admixture8 | 8 | Among groups | 62.7 |
|  |  | Among sites | 12.0 |
|  |  | Within sites | 25.2 |
| admixture9 | 9 | Among groups | 63.5 |
|  |  | Among sites | 11.3 |
|  |  | Within sites | 25.3 |
| admixture10 | 10 | Among groups | 61.8 |
|  |  | Among sites | 12.2 |
|  |  | Within sites | 26.0 |
| admixture11 | 11 | Among groups | 65.1 |
|  |  | Among sites | 8.7 |
|  |  | Within sites | 26.2 |
| admixture12 | 12 | Among groups | 63.9 |
|  |  | Among sites | 9.7 |
|  |  | Within sites | 26.4 |
| admixture13 | 13 | Among groups | 64.7 |
|  |  | Among sites | 8.6 |
|  |  | Within sites | 26.7 |
| Groups | **No. of groups** | **Variance components** | **% of variation** |
| Combined assignment, 12 MUs | 12 | Among groups | 65.7 |
|  |  | Among sites | 8.0 |
|  |  | Within sites | 26.3 |
| Combined assignment, 11 MUs - Salmon + Marys | 11 | Among groups | 65.6 |
|  |  | Among sites | 8.4 |
|  |  | Within sites | 26.0 |
| Combined assignment, 11 MUs - SF Owyhee | 11 | Among groups | 65.0 |
|  |  | Among sites | 8.8 |
|  |  | Within sites | 26.3 |
| Combined assignment, 11 MUs - North Fork | 11 | Among groups | 64.7 |
|  |  | Among sites | 9.1 |
|  |  | Within sites | 26.3 |

**Table S5.** Effective population size (*N*_e_) estimates using a maximum allele frequency of 0.10 (i.e.,

Pcrit = 0.10). *N*_e_ and jackknife confidence intervals (CIs) are corrected for the number of

chromosome pairs in *Rana pretiosa*. The “inf” estimate indicates that the confidence interval

includes infinity.

| Site | Management Unit | Number of loci | Number of individuals | Corrected *N*_e_ | Lower CI | Upper CI |
| --- | --- | --- | --- | --- | --- | --- |
| MERL | Bruneau | 10000 | 12 | 22.8 | 9.5 | 1037.6 |
| MERU |  | 10000 | 12 | 25.2 | 14.7 | 61.9 |
| TEGU |  | 10000 | 12 | 39.3 | 16.2 | inf |
| COCA | Owyhee | 10000 | 12 | 27.2 | 8.7 | inf |
| RICR |  | 10000 | 12 | 48.7 | 23.8 | 508.0 |
| TRCR |  | 10000 | 12 | 24.6 | 9.7 | inf |
| WCSP |  | 10000 | 12 | 57.2 | 29.6 | 343.1 |
| DOGE | South Fork Owyhee | 10000 | 12 | 70.5 | 29.9 | inf |
| WICR |  | 10000 | 12 | 19.2 | 7.0 | inf |
| CACR | Salmon Falls | 4860 | 12 | 6.2 | 2.1 | 30.5 |
| DRCR |  | 2718 | 12 | 17.1 | 8.9 | 51.8 |
| CUCR | Marys | 6229 | 12 | 13.9 | 4.2 | 1019.6 |
| HACR |  | 5848 | 12 | 44.3 | 25.8 | 123.4 |
| EFBE | North Fork | 10000 | 14 | 14.6 | 8.7 | 29.2 |
| MACR |  | 10000 | 12 | 12.4 | 3.8 | 112.9 |
| NFHU |  | 10000 | 12 | 20.6 | 7.1 | inf |
| TACA | Independence | 9122 | 12 | 77.4 | 35.2 | inf |
| COSP | Maggie | 3257 | 12 | 10.7 | 4.0 | 39.5 |
| UMAG |  | 4487 | 11 | 53.0 | 19.5 | inf |
| WILL | Willow | 4495 | 12 | 9.7 | 4.5 | 23.8 |
| WILU |  | 4550 | 11 | 30.5 | 14.5 | 257.2 |
| GRMO | Ruby | 3190 | 12 | 14.7 | 5.2 | 253.1 |
| PEAL |  | 1996 | 12 | 13.6 | 5.6 | 71.8 |
| ARDO | Reese | 771 | 12 | 33.7 | 16.7 | 212.0 |
| CLCR |  | 1057 | 12 | 12.6 | 4.6 | 75.5 |
| INVA |  | 814 | 12 | 8.5 | 3.0 | 27.5 |
| WARN |  | 675 | 12 | 32.5 | 13.8 | inf |
| FAPO | Big Smoky | 698 | 12 | 2.1 | 2.6 | 56.4 |
